# The inhibition of TXNRD1 by methylglyoxal impairs the intracellular control of *Mycobacterium tuberculosis*

**DOI:** 10.1016/j.redox.2025.103741

**Published:** 2025-06-25

**Authors:** Hanxiong Li, Ruining Liu, Gokul Raj Kathamuthu, Radosveta Gencheva, Zhen Gong, Axel Tobias Scholz, Mohammad Alzrigat, Lucia Coppo, Elias S.J. Arnér, Martin E. Rottenberg

**Affiliations:** a Dept Microbiology, Tumor and Cell Biology, Stockholm, Sweden; b Division of Biochemistry, Dept of Medical Biochemistry and Biophysics, Karolinska Institutet, Stockholm, Sweden; c Dept of Selenoprotein Research and National Tumor Biology Laboratory, National Institute of Oncology, Budapest, Hungary

**Keywords:** Tuberculosis, Diabetes, Macrophage, Methylglyoxal, Thioredoxin reductase, NRF2

## Abstract

Type 2 diabetes (DM) is a risk factor for development of tuberculosis (TB). Methylglyoxal (MGO), a reactive carbonyl increased during DM targets diverse macromolecules. Here we discovered that MGO converted the mammalian selenoprotein thioredoxin reductase 1 (TXNRD1) to a NADPH oxidase, activating the NRF2 transcription factor in bone marrow macrophages (BMM). NRF2 signaling hampered the production of immune molecules by BMM, thus allowing intracellular growth of *M. tuberculosis (Mtb)*. The overexpression of NRF2 was sufficient to increase the *Mtb* growth. Several inhibitors of TXNRD1 mimicked the effects of MGO on *Mtb* growth in BMM. MGO and the TXNRD1 inhibitor auranofin also increased the susceptibility of mice to *Mtb* infection. Finally, IFN-γ abrogated the MGO-triggered suppression of the protective responses to *Mtb* in BMM, by epigenetic regulation of immune genes, without impairing NRF2 activation. Thus, metabolic alterations in DM may have a causative role in TB-DM comorbidity, by activating NRF2 responses that impair immune protective responses.

## Highlights

• Risk of developing TB is increased during type 2 diabetes (DM).
• Methylglyoxal, increased in DM, binds to TXNRD1 converting it in a NADPH oxidase.
• ROS produced activates NRF2 responses which hamper macrophage immune defenses and *Mtb* control.
• Specific inhibitors of TXNRD1 also increase the susceptibility to *Mtb* via NRF2 activation.
• IFN-γ abrogates the MGO suppression of protective macrophage responses.

## Graphical abstract

**Figure:**
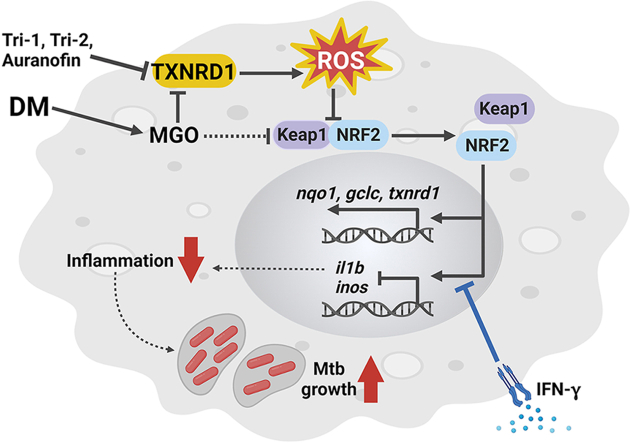

## 1 Introduction

Although only a fraction of individuals infected with *M. tuberculosis* (*Mtb*) develop tuberculosis (TB), in 2021, over 10 million people developed TB, resulting in 1.6 million deaths [1]. The reasons behind the divergent outcomes following infection remain incompletely understood, but epidemiologic studies have revealed a threefold higher risk of developing active TB among patients with type 2 diabetes (DM), regardless of TB endemicity [[2], [3], [4]]. However, the underlying mechanisms behind the DM-TB association are unknown.

Infection occurs when inhaled *Mtb* reach the lung alveoli and are phagocytized by alveolar macrophages [5]. Infected macrophages will recruit phagocytes, including inflammatory macrophages, which contribute to the bacterial control. Phagocytic cells produce oxidants that play a crucial role in clearing the pathogen but can also lead to tissue injury. Hence, regulating oxidative stress is an essential host defense mechanism.

The redox-sensitive transcription factor NRF2 controls the body's antioxidant activities, regulating the expression of genes encoding key components of the glutathione and thioredoxin systems [6,7]. In the cytosol, NRF2 interacts with a Keap1-containing ubiquitin ligase complex, leading to its ubiquitylation and degradation thus preventing its translocation to the nucleus. However, during stress, modifications to Keap1 by electrophilic molecules disrupt the Keap1-NRF2 interaction, activating NRF2 responses [8].

DM is a metabolic disease characterized by high blood glucose levels. Intracellular hyperglycemia causes excessive production of reactive oxygen species (ROS) shunting early glycolytic intermediates into pathogenic molecules such as the reactive carbonyl methylglyoxal (MGO) [9]. MGO mediates rapid non-enzymatic glycation of macromolecules impairing their functions and forming advanced glycation end products (AGEs) [10]. Plasma and tissular MGO levels accumulate abnormally in DM patients and play a prominent role in the pathogenesis of core aspects of DM as shown in clinical and experimental rodent models [[11], [12], [13], [14], [15], [16]].

AGEs activate several stress-induced transcription factors [17], and MGO has been shown to directly bind to Keap1 resulting in the activation of the NRF2 pathway [18]. NRF2 activation has been suggested to impair the early control of *Mtb* growth in alveolar macrophages [19].

In this study we discovered that MGO binds to the thioredoxin reductase 1 (TXNRD1) that catalyses the reduction of the disulphide-reducing protein thioredoxin, converting TXNRD1 into a NADPH oxidase, which subsequently activates NRF2 antioxidant program in BMM. The NRF2 activation also impaired the production of immune molecules that control the intracellular growth of *Mtb*. Altogether, our results suggest that NRF2-mediated responses to the glycolytic by-product MGO can be involved in the elevated risk of developing TB in DM patients.

## 2 Methods

### 2.1 Mice

The animals were housed according to directives and guidelines of the Swedish Board of Agriculture, the Swedish Animal Protection Agency, and the Karolinska Institutet (djurskyddslagen 1988:534; djurskyddsförordningen 1988:539; djurskyddsmyndigheten DFS 2004:4). The study was performed under approval of the Stockholm North Ethical Committee on Animal Experiments permit number 1374–2020. Mice were housed at the Comparative Medicine Biomedicum and the Astrid Fagræus Laboratories, Karolinska Institutet, Stockholm, under specific pathogen-free conditions. All animals used in this study were 7–15 weeks of age mice. In vivo infections were performed in a biosafety level III animal facility.

Macrophages from C57BL/6, *Nrf2*^*−/−*^ [20] and from *Keap1*^*fl/fll*^ *lysm cre* mice were used for *in vitro* experiments. *Keap1*^*fl/fl*^ mice with a loxP-targeted deletion of the *Keap1* gene [21] were crossed with *Lysm cre* transgenic animals in which the cre recombinase expression is driven by the lysozyme M promoter [22], allowing the deletion of the transcription factor in the myeloid lineage. The *Keap1*^*fl/fl*^ mice have been shown to express lower levels of *keap1* [23], so both C57BL/6 and *Keap1*^*fl/fl*^ were used as controls of *Keap1*^*fl/fll*^ *lysm cre* mice. The C57BL/6 congenic strain carrying the differential pan leukocyte marker Ly5.1 and the p25 αβT cell receptor transgenic mice recognizing the p25 epitope of *Mtb* Ag85b were also used.

### 2.2 Infection of mice with *M. tuberculosis*

Mice were infected with 200 CFU *Mtb* Harlingen strain using a nose-only aerosol exposure unit (In-tox Products, New Mexico, USA). The dose indicates the bacteria recovered in lungs 24 h after infection. A 15-ml suspension of 1 × 10^6^ *Mtb* per ml was loaded into a nebulizer, and animals inhaled the bacteria aerosol for 20 min. Mice were sacrificed at indicated time points after infection and bacteria from organ lysates was plated and quantified on Middlebrook 7H11 agar containing 10 % enrichment of oleic acid, albumin, dextrose, catalase, 5 μg amphotericin B per ml and 8 μg/ml polymyxin B grown for at least 3 weeks at 37 °C.

### 2.3 Generation of mouse bone marrow-derived macrophages

Bone marrow cells were flushed from tibia and femurs with PBS, filtered through a 70 μm cell strainer, resuspended in DMEM supplemented with 10 % FCS and 30 % L929 cell-conditioned medium (as a source of macrophage-colony stimulating factor) and incubated for 6 days at 37 °C, 5 % CO_2_. Bone marrow-derived macrophage (BMM) cultures were then washed with PBS, detached with 1 %trypsin, and 5 × 10^5^ cells were seeded to each well at (using 24 well plates). BMM were further incubated for 24 h at 37 °C before infections or treatment with diverse compounds. Confirming previous data [24], BMM were F4/80+, CD11b+, CD11c- and SiglecF-.

### 2.4 Infection of BMM with mycobacteria

*Mtb* H37Rv carrying the green fluorescent protein (GFP)-encoding pFPV2 plasmid [25] were grown in Middlebrook 7H9 (Difco, Detroit, MI) supplemented with albumin, dextrose and catalase and quantified by densitometry. BMM were infected with sonicated bacteria at a multiplicity of infection (MOI) of 2 unless otherwise indicated. After 4 h, cells were washed twice with PBS to remove extracellular bacteria and further incubated for 1–5 days. The infected BMM were then detached using trypsin-EDTA at different times after infection, incubated with live/dead stain (LIVE/DEAD™ Fixable Yellow Dead Cell Stain, Invitrogen), washed with FACS buffer (PBS containing 0.5 % FCS and 0.5 mM EDTA] and fixed with 4 % formaldehyde (Sigma-Aldrich) at room temperature overnight. Data were acquired on a Sony ID7000 spectral cytometer and analysed with FlowJo software (Tree star Inc., Ashland, OR). Cells in the mononuclear gate were ≥95 % live and ≥95 % of live cells are CD11b+, F4/80+. This procedure allowed the exclusion of *Mtb* associated with dead cells, the determination of the frequency of infected cells and the relative levels of intracellular bacteria.

### 2.5 Small interfering RNA gene silencing

Transfections with siRNA were performed as previously described [26] using sequences listed in the Supplementary Table 2. BMM were plated at 5 × 10^5^ cells per ml in 24-well plates overnight. On the day of transfection, the media was replaced with 450 μl DMEM without penicillin/streptomycin or L-929 cell supernatants. For each target gene, two vials were prepared. Optimem (25 μl per well) was added to each tube. 2.5 μl of the cationic lipid lipofectamine RNAimax (InVitrogen) was added to one set of tubes and 50 nM per well short interfering RNA (siRNA) unless otherwise indicated, was added to the second set of tubes. The siRNA was then added to the vial with RNAimax, mixed well by pipetting and incubated for 15 min. 50 μl of the mix was incubated with the BMM cell culture. Twenty-four hours after transfection, cells were infected or incubated with different molecules.

### 2.6 Real time PCR

Total RNA was extracted from lung samples or culture cells using Trizol (Sigma Aldrich) and cDNA was obtained by reverse transcription. Transcripts were quantified by real time PCR as described [27]. Transcripts were quantified using *hprt* as a control house-keeping gene to calculate the ΔCt values for individual samples. The relative number of transcripts was calculated using the 2^-(ΔΔCt)^ method. The primer sequences used are listed in Supplementary Table 1. These values were then used to calculate the relative expression of mRNA in the different conditions (infection and/or treatment) used in tissues and cells.

### 2.7 Western blot

BMM were lysed in RIPA buffer supplemented with protease and phosphatase inhibitor cocktail. Protein concentration was measured using BCA protein assay kit (Biorad) and 15 μg protein was separated on 10 % separating/5 % stacking SDS-polyacrylamide gels. Samples were then transferred onto nitrocellulose membranes (BioRad, Hercules, CA) by electroblotting at 100 V, 250 mA for 80 min. Immunostaining was performed using polyclonal rabbit anti-NRF2 or anti-actin. Membranes were then washed and incubated with horse-radish peroxidase-conjugated polyclonal goat anti-rabbit immunoglobulin and developed using ECL-Plus (Amersham Biosciences, Buckinghamshire, UK) and photographed using a Fuji intelligent dark box II digital camera.

### 2.8 Nitrite determinations

To analyse the concentration of the stable oxidation products of nitric oxide (NO) in the BMM supernatants, the total concentration of nitrite was calculated by performing the Griess reaction as previously described [28]. Briefly, 100 μl of 1 % (w/v) sulfanilamide in 5 % phosphoric acid followed by 100 μl 0,1 % (w/v) N-(1-naphtyl) ethylenediamine HCl was added to 50 μl of samples. After incubation for 10 min RT the absorbance was read at 540 nm.

### 2.9 RNA sequencing

RNA was extracted from BMM, infected or not with *Mtb and* treated with MGO 4 h before infection using Trizol (Sigma Aldrich). The RNA quality was assessed by 2200 TapeStation Instrument (Agilent, Santa Clara, CA). PolyA RNA selection was performed using the Illumina TruSeq RNA Sample Preparation Kit according to the manufacturer's protocol. RNA-seq libraries were prepared and sequenced on the Illumina HiSeq 2000 platform. Pre-processed reads were aligned to the standard mouse reference genome mm10 using the HISAT2 program, and Hypergeometric Optimization of Motif EnRichment (HOMER, http://homer.salk.edu/homer) was used to create the tag directory and count tags in all exons. For the gene expression analysis, unsupervised hierarchical clustering and principal component analysis of genes were performed in Qlucore Omics Explorer 3.2 (Qlucore, Lund, Sweden). Differentially expressed genes were determined by comparing groups using heteroscedastic two-tailed t tests. Multiple testing correction was performed using the Benjamini-Hochberg algorithm with a false discovery rate (FDR) of 1 %. Gene Ontology enrichment analysis (Biological Process, Molecular Functions) or KEGG pathway enrichment was performed with WebGestalt (http://www.webgestalt.org) using default parameters. The raw and processed RNA sequencing data can be found at GSE271061 of the GEO/NCBI repository.

Differential gene expression analysis was performed with R (version 4.3.1) using the Deseq2 package. Only genes with more than 10 raw counts across all samples were included in the analysis. Genes with adjusted p-value less than 0.05 were considered significant. For visualization, the count data was log_2_ transformed and Z-scaled (x-mean(row))/sd(row)). The differentially expressed genes were clustered using k-means clustering and a heatmap was generated using the ComplexHeatmap package in R.

Each dot in the MA map of DEG represents a gene. The log_2_ FPKM, the mean expression amount in the two samples and the log_2_ FC the log value of each gene expression difference between the two samples were plotted.

### 2.10 Thioredoxin reductase activity inhibition assays

The TXNRD enzyme assays were performed in a reaction buffer containing 50 mM Tris pH 7.5 with 2 mM EDTA and 0.1 mg/ml bovine serum albumin. Recombinant human TXNRD1 or *E. coli* TXNRD (15 nM) was added to 0–5 mM MGO or 2 μM TRI-1 spotted on 96-well plates and incubated for 40 min with 0.25 mM NADPH and then 2.5 mM DTNB was added. NADPH consumption was followed at A_340_. For the assays of inhibition of *E. coli* TXNRD, 4 μM E*. coli* thioredoxin was also added to the reaction. TNB formation was followed by reading A_412_ for 15 min using a Spectra Max plate reader. Assays were performed in duplicates using 96-well plates, with activity inferred from absorbance. The experiment was independently repeated two more times in singlets, showing the same results.

### 2.11 Juglone reduction assay

Formation of SecTRAPs was assessed using a juglone reduction assay [29]. MGO or TRI-1 were incubated for 90 min in a reaction containing 300 nM TXNRD1, 250 μM NADPH, and BSA (0.1 mg/ml), completely inhibiting Sec-dependent TXNRD1 function. Reactions were then added to a buffer containing 100 μM juglone and 250 μM NADPH, and NADPH consumption was measured at *A*_340_ for 10–15 min.

### 2.12 TXNRD activity in cell lysates

BMM were washed twice with PBS, then 200 μL of lysis buffer (50 mM Tris-HCl, 2 mM EDTA, 0.15 M NaCl, 1 % Triton X-100, protease (Roche), pH 7.5) was added and cells were scraped, collected into tubes and snap frozen. After three freeze-thaw cycles, cell lysates were centrifuged (13 000 rpm, 15 min), supernatants were collected, and protein concentrations measured using Pierce bicinchoninic acid assay (BCA) protein assay kit (Thermo Fischer Scientific) according to the manufacturer's protocol. To measure TXNRD activities, the endpoint TXN-dependent insulin reduction assay was used, as described previously [29]. In short, 7.5–9 μg total protein from the cell lysate was incubated with 0.16 mM human insulin (Sigma), 0.33 mM NADPH (Saveen Werner) and 16 μM human TXN in TE buffer (50 mM Tris-HCl, 2 mM EDTA, pH 7.5) at a total volume of 50 μL and was then incubated at 37 °C. After 0, 15 and 30 min, 10 μL aliquots were taken and combined with 6 M guanidine-HCl and 2.5 mM DTNB in 96-well plates (PerkinElmer), whereupon the absorbance at 412 nm was measured using microplate spectrophotometer (TECAN).

### 2.13 Total glutathione and GSH/GSSG ratio estimation

Samples were tested for protein concentration and one aliquot was deproteinized with 6 % sulfosalicilic acid. After 20 min at 4 °C, samples were centrifuged at 15800 × *g* for 10 min at 4 °C. Supernatants was utilized for total glutathione estimation performed as previously described [30]. For measurement of oxidized glutathione, samples supernatants were immediately treated with 2 % 2-vinylpyridine for 40 min before performing the assay in order to sequestrate reduced glutathione and be able to quantify the oxidized glutathione.

### 2.14 Determination of H_2_O_2_ in culture supernatants

After incubation with compounds or infection, the cell culture medium was changed to PBS and cells were further incubated for 90 min. Then, 100 μl supernatant from each well was mixed with 20 μl of reaction mix with a final concentration of 500 μM Amplex Red and 1 U/ml horseradish peroxidase, incubated for 30 min before the fluorescence was monitored (560 nm excitation/590 nm emission).

### 2.15 Cell ROX

To assess the oxidative stress levels, BMM were incubated with 5 μM CellROX-Deep-Red (Invitrogen) for 30 min, washed with PBS, trypsinized and resuspended in PBS, before FACS analysis. The cell-permeant dye is non-fluorescent while in a reduced state and exhibits bright fluorescence upon oxidation by ROS, with absorption/emission at 644/665 nm.

### 2.16 BMM viability XTT assay

BMM viability was assayed by the XTT assay (Roche Diagnostics), based on the conversion of the XTT tetrazolium salt to formazan. BMM were washed once with PBS, followed by the addition of 100 μl of fresh media containing 0.2 mg/ml XTT and 0.43 μg/ml menadione to each well. Empty wells containing only the XTT-menadione solution were included as blanks. The plate was incubated for 3 h at 37 °C 5 % CO_2_. Then absorbance was measured at 450 nm and 690 nm using a plate reader (Thermo Scientific™ Multiskan™ GO Microplate Spectrophotometer). The 690 nm absorbance background was subtracted from the 450 nm reading, and the values from the blank wells were subtracted from the sample wells to obtain the final absorbance values. The percentage of cell survival was calculated by comparing the absorbance of treated samples to the untreated control with the same genotype.

### 2.17 Malondyhaldehyde (MDA) determination

To determine lipoperoxidation in *Mtb* infected and/or treated BMM, a fluorometric TBARS assay for MDA detection (Cayman Chemical) was used. 1.5 × 10^6^ BMM were detached from 6 well plates, pelleted and lysed in 50 μl RIPA buffer. The TBARS assay was performed as described by the manufacturer in 96 black well plates, using MDA as standard. Absorbance was measured using a Multiskan™ GO Microplate Spectrophotometer at 530 nm.

### 2.18 IL-1β measurement in culture supernatants

The concentration of IL-1β in BMM supernatants was quantified by ELISA, according to the manufacturer's instructions (BioLegend ELISA MAX™ Standard Set Mouse IL-1β).

### 2.19 Flow cytometry of lung cells

Lungs were removed, mechanically minced into small pieces and digested with 3 mg/ml Collagenase D and 30 μg/ml DNase I for 1 h at 37^o^C, and single-cell suspensions prepared by filtering lung tissue through 70 μm nylon cell strainers. To enrich the suspension in lymphocytes, cells were loaded into an isotonic 40–70 % Percoll density gradient and centrifuged for 30 min at room temperature. Cells in the gradient interphase were collected and washed before further labelling. Cell suspensions were incubated with live/dead stain (LIVE/DEAD™ Fixable Aqua Dead Cell Stain Kit, Invitrogen™). Then CD16/CD32 blocking antibodies (BD) and the fluorophore conjugated antibody cocktails (Supplementary Table 4) were introduced and incubated for 30 min on ice. Cells were then washed with PBS, resuspended and fixed with 2 % paraformaldehyde solution in PBS. To determine the expression of intracellular IL-1β and iNOS, cell suspensions prepared as described above were fixed and permeabilized using the eBioscience™ Intracellular Fixation & Permeabilization Buffer Set according to the manufacturer's protocol and stained with specific antibodies for iNOS and IL-1β. Data were acquired on a Sony ID7000 full spectral flow cytometer and analysed with FlowJo software (Tree star Inc., Ashland, OR). Examples of the supervised gating strategies used are shown (Supplementary Fig. 6H). The unsupervised nonlinear dimensionality reduction algorithm tSNE platform from FLowJow was used for visualizing the high dimensional flow cytometry data sets in a dimension-reduced data space. tSNE coordinates were assigned to each cell in each sample using a concatenated file containing 150000 F4/80+ cells per group.

### 2.20 Microscopy

BMM were fixed with 4 % PFA and incubated with stained with anti-NRF2 (1:100; 12721T; Cell Signaling Technologies) antibody in 0.1 % Tween 20, 5 % BSA and 5 % donkey serum PBS for 1 h. Cells were then washed with 1 × PBS-T and further incubated with Rhodamine Red™-X (RRX) AffiniPure™ Donkey Anti-Rabbit IgG (H + L) (Jackson ImmunoResearch) 1:100 for 1 h at room temperature, followed by staining with DAPI for 15 min and washed and mounted in Fluoromount G (Southern Biotech). Slides were analysed with a Leica epi-fluorescence microscope using 40 × objective. Fluorescence intensity was quantified in three to five random fields of view per coverslip with the Cell Profiler software pipeline. Briefly, DAPI channel was segmented using the “IdentifyPrimaryObjects” function to detect nuclei, and Rhodamine Red intensity was then measured.

### 2.21 Chromatin immunoprecipitation-qPCR

Chromatin immunoprecipitation (ChIP) was conducted using the OneDay ChIP kit (Diagenode) following the manufacturer's protocol. In brief, BMM were treated with 1 % (wt/vol) formaldehyde to cross link DNA-protein complexes for 10 min at RT and subsequently quenched with 125 mM glycine in PBS. After washing with PBS, BMM were detached and suspended in lysis buffer (20 mM Tris-HCl, 10 mM EDTA, 0.15 M NaCl, 0.5 % Triton X-100, 30 mM sodium pyrophosphate, 0.5 % sodium deoxycholate salt). The lysates were then sonicated (Misonix Sonifier) 6 rounds of 10 cycles of 30 s ON and 30 s OFF pulses to shear DNA into approximately 500 bp fragments. The nuclear proteins/DNA were labelled with antibodies against NRF2 (Cell Signaling technology; D1Z9C; 1:250 dilution, RNA polymerase II (Upstate; CTD4H8; 1:250 dilution) or IgG control and precipitated using antibody binding beads. After extensive washing of the enriched antibody-DNA complex cross-linking was reversed by heating and proteinase K treatment and DNA further purified in a column to remove remaining proteins. The enrichment of specific sequences was analysed by qPCR using primers (Supplementary Table 3) that flanked regions containing putative binding sites to the NRF2 or the Pol II polymerase as shown in a NRF2 ChIP-Seq DNA data from mouse macrophages [31]. The results are calculated as the % recovery of the input (samples after reverse crosslinking not undergoing immunoprecipitation). Highly expressed genes, non-transcribed genes or sequencing not binding to NRF2 were used for positive and negative controls.

### 2.22 Formaldehyde-assisted isolation of regulatory elements (FAIRE)

The FAIRE was performed as previously described [32]. Briefly, 500‐bp DNA fragments were prepared by sonication as in ChIP protocol above. The sample was then subjected to phenol/chloroform extraction, whereby nucleosome-depleted DNA partitioned into the aqueous phase while nucleosome-bound DNA remained at the interface. After three rounds of extraction, the DNA was precipitated, purified, reverse crosslinked, and quantified for qPCR using primers in Supplementary Table 3. The regions amplified were selected after analysis of the genome browser tracks (Supplementary Fig. 5O). The results were expressed as the recovery ratio towards the input (samples that were reverse crosslinked without phenol/chloroform extraction) and normalized to the untreated control. The accession number in the repository, references and the treatments used for macrophage stimulation depicted in the genome tracks (obtained using integrative genomics viewer) are shown in the Supplementary Table 5.

### 2.23 Statistical analysis

All *in vitro* assays were performed at least in biological triplicates and independently repeated twice or more. Differences in bacterial counts in the lungs of infected mice were calculated using the non-parametric Mann-Whitney's *U* test. Differences in cytokine transcripts, lactate, nitrites and CFU were measured by unpaired Student's *t*-test considering unequal variances (Welch's test) and by one way ANOVA using the Welch's adjustment when comparing three or more groups. In this case a two-way ANOVA was used when more than two parameters were analysed (i.e. time after infection and treatments). Multiple comparisons were corrected by False Discovery Rate method. The statistical tests were performed using the Prism software (GraphPad, La Jolla, CA).

## 3 Results

### 3.1 MGO promotes the expression of antioxidant genes and impairs immune response gene accumulation in *M. tuberculosis*-infected macrophages

**Fig. 1:**
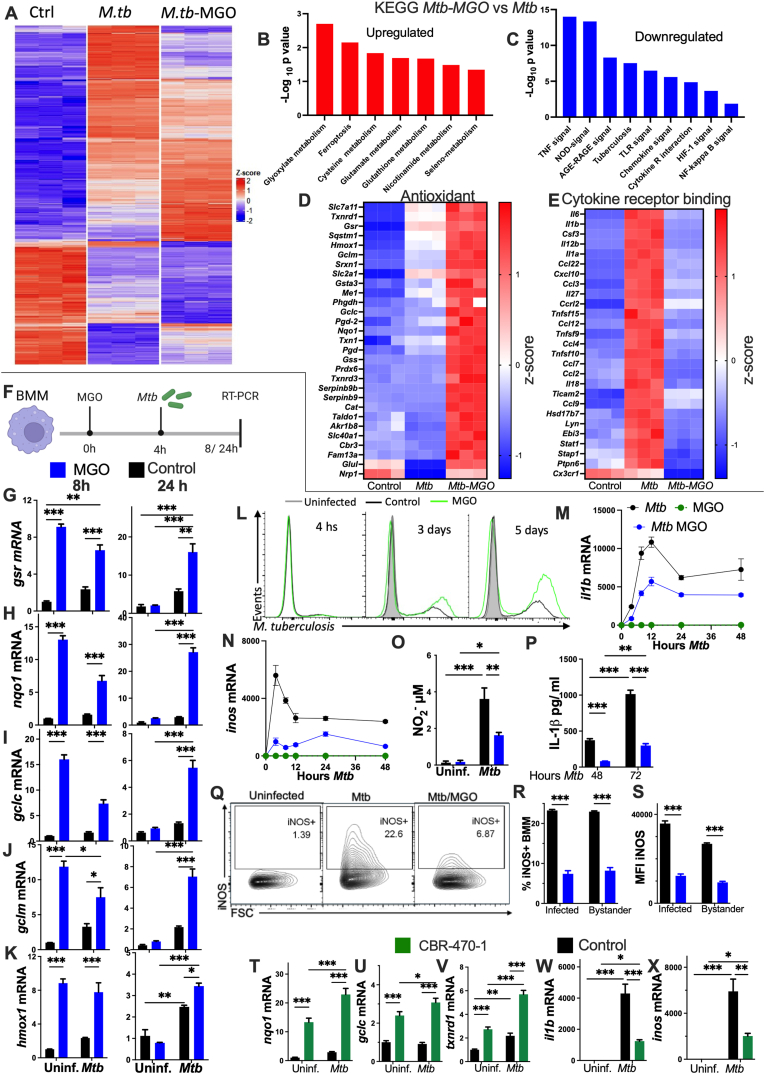
MGO impairs the expression of inflammatory genes and stimulates the expression of antioxidant genes in *M. tuberculosis* infected BMM **(A)** RNA seq was performed in triplicate independent cultures of BMM treated or not with 200 μM MGO and infected 4 h after with *Mtb* MOI of 3:1. RNA was extracted 4h after infection. Differentially expressed genes (DEGs) were defined were set as fold change (FC)≥2 and FDR<0.01 between groups. The heatmap of DEG were generated with log_2_ transformed and Z-scaled (normalizing the individual data in each row by subtracting the row mean and then dividing by row standard deviation). The DEGs were clustered using k-means clustering and a heatmap was generated using the Complex Heatmap package in R. **(B and C)** The log_10_ p value of the most enriched terms after KEGG pathway analysis of *Mtb-MGO vs Mtb* upregulated or downregulated DEGs were obtained by using the WebGestalt software. **(D and E)** The heat maps showing DEGs in the antioxidant and cytokine receptor signaling pathways were normalized by subtracting the log_2_ values to the mean log_2_ value for all samples for each gene. **(F)** BMM were treated with 200 μM MGO and infected 4 h after with *Mtb at* a MOI of 5:1. Total RNA was extracted 8 or 24 h after MGO treatment. **(G**–**K)** The fold increase of *gsr*, *nqo1*, *gclc*, *gclm* and *hmox1* mRNA normalized to the *hprt* mRNA levels in the same sample and to untreated controls were determined by RT-PCR. **(L)** BMM were treated or not with 200 μM MGO and infected 4h after with *Mtb*-GFP at a MOI of 3:1. Representative histograms of *Mtb*-GFP in BMM at different time points after infection are shown. **(M and N)** BMM treated or not with MGO and 4h after infected with *Mtb*. The levels of *il1b* and *inos* mRNA were measured in total RNA extracted at the indicated time points after infection. **(O)** The nitrite (NO_2_^−^) concentration in supernatants of BMM treated or not with MGO was measured by Griess assay 72 h after infection with *Mtb.* **(P)** IL-1β levels were measured by ELISA in the supernatants of BMM treated or not with MGO after infection with *Mtb.* **(Q)** Representative contour plots of iNOS expression in MGO-treated and control BMM 72h after *Mtb* infection, as well as in uninfected BMM are depicted. **(R and S)** The frequency of iNOS expressing cells and the MFI among infected and bystander uninfected BMM in the same culture are shown. **(T**–**X)** BMM treated with 5 μM CBR-470-1 for 4h before *Mtb* infection. The increase of *nqo1*, *gclc*, *txnrd1*, *il1b* and *inos* mRNA 24h after infection is shown. **(G-K, M-P, R,S, T-X)** The mean ± SEM of triplicate independent cultures. **(G-K, O,P, R,S, T-X)** Differences between groups are significant (∗p ≤ 0.05; ∗∗p ≤ 0.01 and ∗∗∗p ≤ 0.001 2-way ANOVA test).

We have previously shown that high glucose levels and MGO impaired the control of *Mtb* in BMM [33]. We first compared the transcriptome profiles of *Mtb* infected BMM treated with 200 μM MGO with untreated controls. The heatmap, Pearson's correlation and PCA analysis showed a differential clustering of uninfected, *Mtb* and *Mtb-MGO*-treated BMM (Fig. 1A and Supplementary Fig. 1A and B). We found 3606 differentially expressed genes (DEGs) between uninfected and *Mtb* BMM and 1699 DEGs comparing *Mtb-MGO vs Mtb* BMM, 2/3 of these DEGs downregulated (Supplementary Fig. 1C–F). The KEGG analysis showed that genes in the glyoxalate, glutathione, ferroptosis, and selenocompound metabolism were increased comparing *Mtb-MGO* vs *Mtb* BMM (Fig. 1B), while innate immune responses genes were decreased in *Mtb*-*MGO* vs *Mtb* BMM (Fig. 1C) as shown for individual transcripts in these gene pathways (Fig. 1E and Supplementary Fig. 1G–I). Several antioxidant genes increased in *Mtb-MGO* vs *Mtb* BMM samples are controlled by NRF2 and regulate glutathione and thioredoxin production and regeneration (Fig. 1D). Thus, immune responses decreased while cytoprotective response transcripts increased in *Mtb*-MGO BMM.

We next validated and extended the transcriptome results by showing that incubation of BMM with MGO increased the levels of NRF2-regulated *gsr, nqo1 gclc, gclm* and *hmox1* at 8 but not 24 h after MGO treatment (Fig. 1F–K and Supplementary Fig. 1J and K). The levels of the antioxidant transcripts were also increased in *Mtb-*MGO BMM as compared to either the *Mtb*-BMM or MGO-treated uninfected BMM when determined 24 h after infection (Fig. 1G–K). The levels of total and reduced glutathione were also increased in *Mtb*-MGO vs. *Mtb*-BMM (Supplementary Fig. 1L and M). Increased NRF2 was detected in BMM lysates at 8 and 24 h after infection of *Mtb* and increased when incubating BMM with MGO (Supplementary Fig. 1N).

Incubation with 200 μM MGO increased the frequency of infected cells and the intracellular bacterial levels in BMM at 2, 3 and 5 days after infection with GFP labelled *Mtb*, while bacterial uptake in *Mtb*-MGO vs. *Mtb* were similar (Fig. 1L and Supplementary Fig. 1O). BMM treated with 50 or 100 μM MGO also showed increased *Mtb* intracellular levels after infection (Supplementary Fig. 1P and Q).

iNOS and IL-1β mediate *Mtb* intracellular growth control [34,35]. The accumulation of *il1b* and *inos* mRNA, intracellular IL-1β and iNOS proteins, and IL-1β and nitrite (the oxidation product of NO) in BMM supernatants increased after *Mtb* infection, and was lower in *Mtb*-MGO than in *Mtb-*BMM (Fig. 1M−S and Supplementary Fig. 1R and S). The expression of iNOS was higher in both *Mtb* infected and bystander (non-infected BMM in the same culture) as compared with uninfected BMM, while bystander and infected *Mtb-*MGO BMM showed lower iNOS levels than *Mtb-*BMM (Fig. 1Q–S).

We next studied whether endogenously generated MGO could also affect the outcome of *Mtb* in BMM. BMM were treated with CBR-470-1, an inhibitor of the glycolytic phosphoglycerate kinase 1 (PGK1) that increases the triosephosphate levels, resulting in the accumulation of MGO [18] (Supplementary Fig. 1T). BMM were incubated with 5 μM CBR-470-1 4h before infection with *Mtb*. We found that NRF2-regulated transcripts in both *Mtb*-infected and uninfected BMM were increased after treatment with CBR-470-1 (Fig. 1T–V and Supplementary Fig. 1U and V). The levels of *il1b* and *inos* mRNA decreased in *Mtb*-infected CBR-470-1-treated BMM as compared to untreated, infected controls (Fig. 1W and X).

Intracellular high glucose levels during diabetes may result in the generation of MGO [9]. Whether incubation of BMM in medium containing high glucose levels can reproduce some the responses regulated by MGO was then tested. BMM were incubated with either 5 or 50 mM glucose before and during the infection with *Mtb*. BMM cultured in 50 mM glucose showed higher intracellular *Mtb* levels 5 days after infection than those cultured in low glucose (Supplementary Fig. 2A and B). Incubation with high levels of glucose also resulted in increased levels of NRF2-regulated transcripts such *nqo1, txnrd1* and *gclc* and diminished titters of *il1b* and *inos* mRNA in *Mtb*-infected BMM (Supplementary Fig. 2C–G).

The effect of MGO on human macrophages was then tested. Incubation with MGO increased the frequency of infected THP-1 macrophages as well as the intracellular bacterial levels (Supplementary Fig. 2H and I). Increased levels of *NQO1* and *TXNRD1* mRNA were observed in THP1-infected or uninfected cells 8h after MGO administration (Supplementary Fig. 2J and K). *IL1B* and *INOS* mRNA in THP-1 macrophages treated with MGO were lower (Supplementary Fig. 2L and M). Thus, the responses to MGO in human THP-1 cells resemble those observed in murine BMM.

Thus, addition or endogenously produced MGO increases NRF2 target molecules and hampers the expression of infection-induced immune molecules and the intracellular control of *Mtb* growth in BMM and in a human macrophage cell line.

### 3.2 NRF2 mediates the impaired control of *M. tuberculosis* in MGO-treated BMM

The role of NRF2 in the regulation of *Mtb* infection by MGO was then investigated by silencing the *nrf2* gene. A Cy5 labelled control siRNA was detected several days after BMM transfection (Supplementary Fig. 3A), and *nrf2* silencing decreased the *nrf2* mRNA accumulation as well as NRF2 protein expression in *Mtb*-MGO BMM or uninfected controls (Supplementary Fig. 3B–D).

**Fig. 2:**
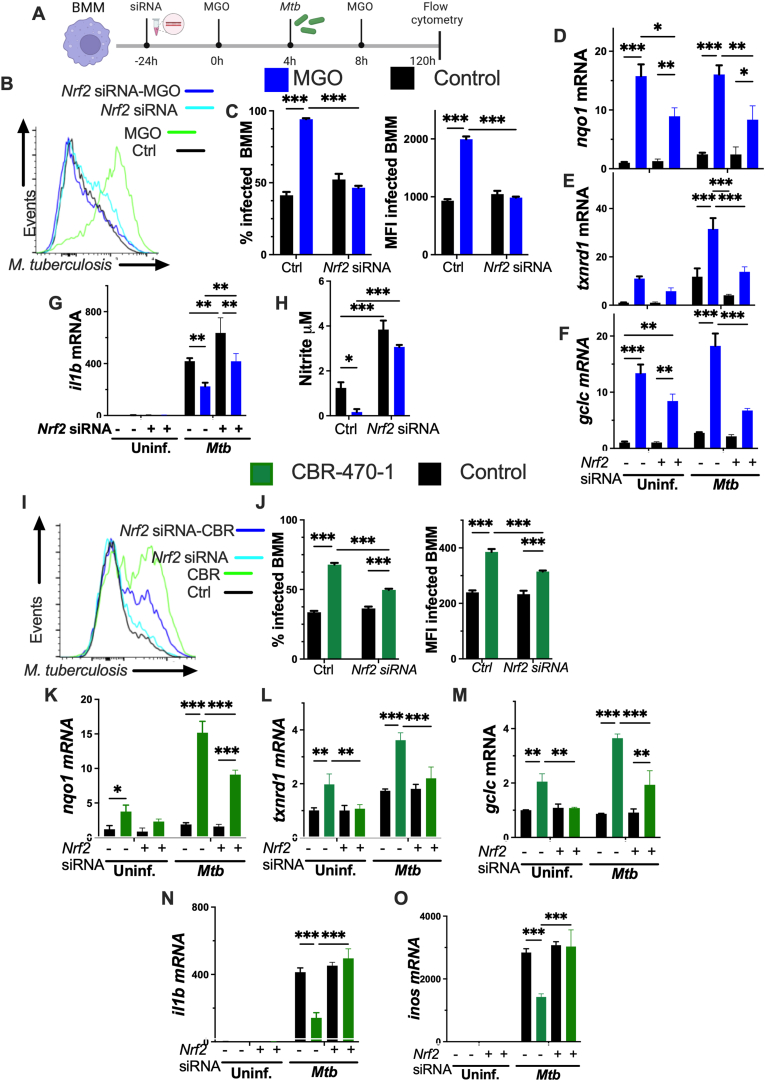
NRF2 mediates the impaired control of *M. tuberculosis* in MGO-treated BMM **(A)** BMM were transfected with *Nrf2* or control siRNA 24h before treated with MGO or left untreated. 4 h after MGO treatment BMM were infected with *Mtb-*GFP*.* MGO was replenished 4h after infection. The *Mtb*-GFP levels in BMM were determined 120 h later. **(B and C)** Representative histogram, the percentage of infected BMM and the *Mtb*-GFP MFI gated on infected cells 5 days after infection. **(D**–**G)** BMM transfected with *Nrf2* or control siRNA and 24 h after were incubated with MGO. 4 h after treatment, groups of BMM were infected with *Mtb.* The levels of *nqo1*, *txnrd1* and *il1b* mRNA were determined 4h after infection. **(H)** The nitrite concentrations were determined by Griess assay in the supernatants of BMM cultures 5 days after *Mtb* infection. **(I and J)** BMM transfected with *Nrf2* or control siRNA, were treated with CBR-470-1 24h after transfection. Four hours after CBR-470-1 treatment, BMM were infected with *Mtb*. A representative histogram, the percentage of infected BMM and the *Mtb*-GFP MFI gated on infected cells, determined 5 days after infection. **(K–O)** BMM transfected with *Nrf2* or control siRNA and 24 h after were incubated with CBR-470-1. 4 h after treatment, BMM were infected with *Mtb. Nqo1*, *txnrd1, gclc, il1b* and *inos* mRNA levels were determined 24h after infection. **(C–O)** The mean fold transcript increase ± SEM in triplicate BMM cultures is depicted. Differences are significant at ∗p ≤ 0.05, ∗∗p ≤ 0.01 and ∗∗∗p ≤ 0.001 2-way ANOVA test.§.

*Mtb* levels in MGO-treated BMM decreased when transfected with *Nrf2* siRNA as compared to control siRNA, measured 5 days after infection (Fig. 2A). The transfection with *Nrf2* siRNA did not alter bacterial levels in MGO-untreated BMM (Fig. 2B and C). The uptake of *Mtb,* as measured at 4h after infection, in *Nrf2* siRNA and control siRNA transfected BMM treated or not with MGO was similar (Supplementary Fig. 3E). The transfection with *Nrf2* siRNA reduced the accumulation of NRF2-regulated transcripts in MGO-treated BMM, infected or not with *Mtb* (Fig. 2D–F). Moreover, the expression of *il1b* mRNA and the accumulation of nitrite in supernatants, which diminished after MGO incubation of *Mtb* infected BMM, increased in MGO-treated BMM transfected with *Nrf2* siRNA (Fig. 2G and H).

Incubation of BMM with CBR-470-1 increased the intracellular titres of *Mtb* measured 5 days after infection, and *Nrf2* siRNA transfection decreased the intracellular levels of *Mtb* in CBR-470-1-treated BMM (Fig. 2I and J), while the uptake of *Mtb* in BMM transfected or not with *Nrf2* siRNA was similar (Supplementary Fig. 3F). The transfection with *Nrf2* siRNA also impaired the expression of NRF2-dependent transcripts in CBR-470-1-treated BMM, infected or not with *Mtb* (Fig. 2K–M). Moreover, *il1b* and *inos* mRNA levels that were lessened by pre-incubation of *Mtb* BMM with CBR-470-1, increased when BMM were transfected with *Nrf2* siRNA (Fig. 2N and O).

Confirming the results obtained by *Nrf2* siRNA transfection, incubation of BMM with ML385, a small molecule that binds and inhibits NRF2 activity [36], diminished *Mtb* levels in BMM treated with MGO when measured 5 days after infection, but did not change bacterial levels in MGO-untreated BMM, or the bacterial uptake (Supplementary Fig. 3G–I). ML385 incubation reduced the levels of *nqo1* mRNA in MGO-treated BMM at 4 and 24h after infection (Supplementary Fig. 3J and K).

Altogether, the addition or the endogenous production of MGO by BMM results in deficient control of intracellular *Mtb* growth through pathways promoting NRF2-activation.

### 3.3 NRF2 stabilization increases the intracellular levels of *M. tuberculosis*

**Fig. 3:**
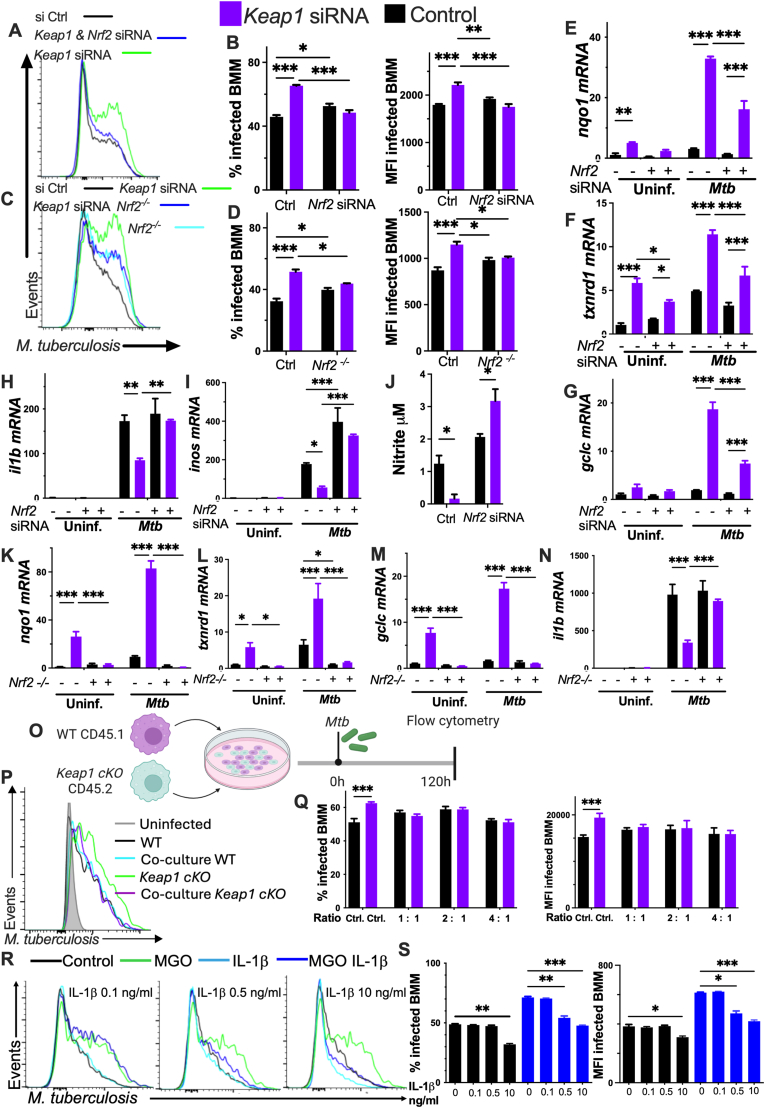
NRF2 stabilization increases the intracellular levels of *M. tuberculosis* **(A and B)** BMM were transfected with *Nrf2, Keap1* and/or control siRNA 24h before infection with *Mtb-*GFP. A representative histogram, the mean percentage of infected BMM and the mean *Mtb*-GFP MFI gated on infected cells determined 5 days after infection in triplicate cultures are shown. **(C and D)***Nrf2*^*−/−*^ or WT BMM were transfected with *Keap1* and/or control siRNA 24h before infection with *Mtb-*GFP. A representative histogram, the mean percentage of infected BMM and the mean *Mtb*-GFP MFI gated on infected cells 5 days after infection ± SEM in triplicate independent cultures are shown. **(E**–**I)** BMM transfected with *Nrf2, Keap1* and/or control siRNA, were infected with *Mtb* or remained uninfected 24 h after transfection. The relative levels of *nqo1*, *txnrd1, gclc, il1b* and *inos* mRNA were determined 24h after infection by RT-PCR. **(J)** The nitrite concentrations were determined by Griess assay in the supernatants of BMM cultures 5 days after *Mtb* infection. **(K–N)***Nrf2*^*−/−*^ and WT BMM were transfected with *Keap1* and/or control siRNA and infected with *Mtb* 24 h after transfection. The fold increase of *nqo1*, *txnrd1*, *gclc* and *il1b* mRNA 24h after infection are depicted. **(O)***Keap1*^*fl/fl*^*Lysm cre (Keap1 cKO)* (CD45.2+) and WT (CD45.1+) BMM were co-cultured at different ratios (or cultured individually) at a final 10^6^ cells/ml density and infected with *Mtb-*GFP. **(P and Q)** A representative histogram, the percentage of infected BMM and the *Mtb-*GFP MFI, were determined 5 days after infection after gating in CD45.1 or CD45.2+ BMM. **(R and S)** BMM treated with MGO for 4 h were infected with *Mtb-*GFP. Four h after infection BMM were incubated with the indicated concentrations of recombinant murine IL-1β, which was further supplemented every day. A representative histogram, the percentage of infected BMM and the *Mtb*-GFP MFI gated on infected cells at 5 days after infection are shown. **(A**–**N)** The mean ± SEM (n = 3 independent cultures per group) are depicted. **(A**–**K)** Differences with control groups are significant at ∗p ≤ 0.05, ∗∗p ≤ 0.01 and ∗∗∗p ≤ 0.001 two-way ANOVA test. (**Q and S**) Differences are significant at p ≤ 0.05, ∗∗p ≤ 0.01 and ∗∗∗p ≤ 0.001, one-way ANOVA.

To study if overexpression of NRF2 is sufficient to hamper the intracellular control of *Mtb*, BMM were transfected with *Keap1 siRNA. Keap1* siRNA transfected BMM showed increased *Mtb* levels measured 5 days after infection. Instead, BMM transfected with both *Keap1* and *Nrf2* siRNA showed similar *Mtb* levels as control or *Nrf2* siRNA treated BMM (Fig. 3A and B). In line with this, co-transfection of *Nrf2*^*−/−*^ BMM with *Keap1* siRNA also failed to increase the intracellular bacterial load in BMM (Fig. 3C and D). *Nrf2* and/or *Keap1* siRNA transfected WT or *Nrf2*^*−/−*^ BMM showed similar uptake of *Mtb* (Supplementary Fig. 3L and M).

The levels of NRF2-controlled transcripts were increased in *Keap1* siRNA transfected-, *Mtb*-infected BMM, while BMM co-transfected with *Keap1* and *Nrf2* siRNA BMM showed diminished the levels of the antioxidant transcripts as compared to WT BMM treated with *Keap1* siRNA alone (Fig. 3E–G). The *Keap1* siRNA transfected, *Mtb-*infected BMM showed diminished *il1b* and *inos* mRNA titters and nitrite levels in the supernatant as compared to those of controls, while co-transfection with *Nrf2* siRNA reconstituted the impaired production of inflammatory molecules (Fig. 3H–J).

*Keap1* siRNA transfected *Nrf2*^*−/−*^ BMM showed lower antioxidant and higher inflammatory levels when compared to *Keap1* siRNA transfected WT BMM (Fig. 3K–N).

Then, whether a decreased production of protective soluble molecules accounted for the increased *Mtb* load in the NRF2-activated BMM was studied. *Keap1* ^*fl/fl*^ *lysm cre* CD45.2+ BMM (*Keap1 cKO*) were cocultured at different ratios with WT CD45.1+ BMM and infected with *Mtb* (Fig. 3O). The *Keap1* *cKO* BMM showed increased levels of NRF2-controlled and reduced *keap1* transcripts as compared with *Keap1*^*fl/fl*^ or WT BMM controls (Supplementary Fig. 3N–P). *Keap1* *cKO* showed higher *Mtb* growth than WT BMM when cultured separately, whereas the bacterial load in cocultured *Keap1* *cKO* and WT BMM were similar (Fig. 3P and Q), suggesting that soluble factors released by WT cells reconstituted *Mtb* growth control in *Keap1 cKO* BMM.

To further validate this finding, whether diminished IL-1β production by MGO-treated BMM accounted for the impaired intracellular *Mtb* control was tested. While the addition of 10 ng/ml IL-1β decreased *Mtb* levels in both, *Mtb* and *Mtb*-MGO BMM, the incubation with 0.5 ng/ml IL-1β, a concentration corresponding to that in supernatants from *Mtb*-BMM (Fig. 1P), reduced the bacterial levels only in *Mtb-*MGO BMM. The *Mtb* titers in MGO-*Mtb* or *Mtb* BMM incubated with 0.1 ng/ml IL-1β were similar to those in IL-1β-untreated controls (Fig. 3R and S).

Thus, we suggest that a diminished release of protective immune molecules by BMM accounts for the NRF2-mediated hampered bacterial growth control.

### 3.4 Thioredoxin reductase mediates the deficient control of *M. tuberculosis*-growth by MGO-treated BMM

**Fig. 4:**
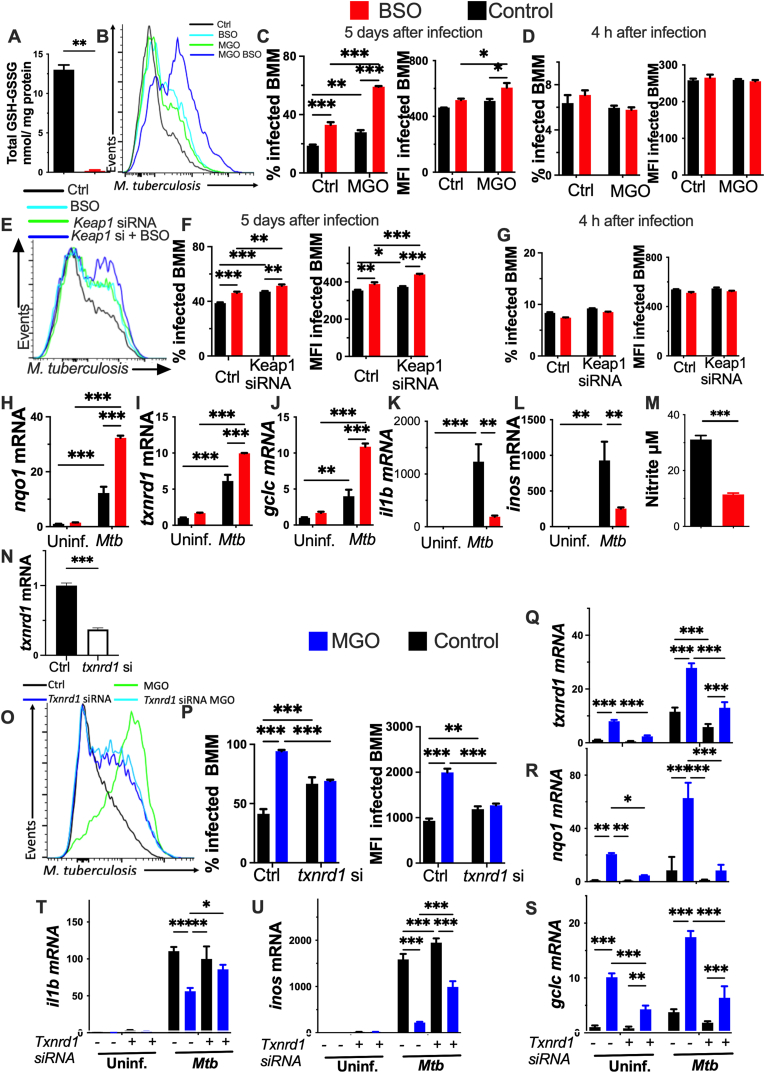
The MGO-induced NRF2 activation in BMM is mediated by TXNRD1 **(A)** The mean total glutathione (GSH + GSSG) nmoles per mg protein in lysates from BMM 24 h after treatment with BSO was measured by the glutathione reductase-coupled DTNB reduction assay in a system with NADPH. **(B**–**D)** BMM were incubated with 200 μM MGO and/or 500 μM BSO, 4h and 1h before infection with *Mtb-*GFP respectively. A representative histogram (at 5 dpi), the percentage of infected BMM and the *Mtb-*GFP MFI in infected cells 4h and 5 days after infection are shown. **(E**–**G)** BMM were transfected with *Keap1* and/or control siRNA 24h before treatment with BSO, and 1 h later were infected with *Mtb-*GFP*.* A representative histogram (at 5 dpi), the percentage of infected BMM and the *Mtb*-GFP MFI in BMM determined 4 h and 5 days after infection are shown. **(H**–**L)** BMM were treated with BSO or left untreated 1 h before infected with *Mtb.* The levels of *nqo1*, *txnrd1*, *gclc*, *il1b* and *inos* mRNA were determined by real time PCR 24h after infection. **(M)** The nitrite titters in supernatants of BMM treated with BSO were measured by Griess assay 5 days after infection with *Mtb.* Differences are significant at ∗p ≤ 0.05 unpaired Student's *t*-test with Welch's correction. **(N)** BMM were transfected for 24h with control or *Txnrd1* siRNA and the *txnrd1* mRNA levels detected by RT-PCR. **(O and P)** BMM were transfected with *Txnrd1* or control siRNA, treated with MGO 24h after and infected with *Mtb-*GFP 4h after MGO-treatment. A representative histogram, the percentage of infected BMM and the MFI gated on infected cells 5 days after infection are shown. **(Q**–**U)** BMM were transfected with control or *Txnrd1* siRNA and treated 20h after with MGO. 4 h after treatment BMM were infected with *Mtb*. The *txnrd1*, *nqo1, gclc, il1b* and *inos* mRNA levels were determined by real time-PCR 24h after infection. **(A**–**U)** The mean fold transcript increase ± SEM in triplicate independent BMM cultures is depicted. **(C-L, P–U)** Differences are significant by 2-way ANOVA, and **(A, M and N)** Student's *t*-test with Welch correction at ∗p ≤ 0.05, ∗∗p ≤ 0.01 and ∗∗∗p ≤ 0.001.

The role of the NRF2-dependent antioxidant molecules in the increased intracellular *Mtb* levels in MGO-treated BMM was next addressed. Glutathione, the most abundant intracellular low molecular Cys-containing molecule, has an important role in the protection against ROS [37]. Buthionine sulfoximine (BSO) inhibits γ-glutamylcysteine ligase, the rate-limiting enzyme of glutathione synthesis [38]. Here, the incubation with BSO, depleted the intracellular glutathione and increased *Mtb* growth in BMM (Fig. 4A–C). Moreover, BSO-treated BMM incubated with MGO or transfected with *Keap1 siRNA* showed higher bacterial levels than those only treated with either BSO (Fig. 4B,C,E and F). The treatment with BSO did not alter the bacterial uptake (Fig. 4D and G). BMM incubated with BSO showed increased levels of antioxidant transcripts and decreased the levels of *il1b* and *inos* mRNA in *Mtb* infected BMM (Fig. 4H–L). Nitrite levels were diminished in BSO-treated BMM as compared to untreated controls (Fig. 4M). Thus, the glutathione synthesis is not required for the processes leading to diminished bacterial control in MGO-treated BMM.

The thioredoxin system is another NRF2 target, with the highly chemically reactive selenoprotein thioredoxin reductase 1 (TXNRD1) that catalyses the reduction of thioredoxin [39,40]. To address the potential role of TXNRD1 in the MGO-mediated impairment of *Mtb* control in BMM, the *txnrd1* gene was targeted by using *si*RNA. The levels of *txnrd1* transcripts, either before or after MGO and *Mtb* treatment, were diminished after *Txnrd1* siRNA transfection (Fig. 4N and Q). Infected MGO-treated BMM showed lower bacterial levels when transfected with *Txnrd1* siRNA (Fig. 4O and P). The expression of NRF2 target genes decreased in *Txnrd1* siRNA transfected and MGO treated BMM infected or not with *Mtb* (Fig. 4R and S). Instead, the titters of *il1b* and *inos* mRNA were increased in *Txnrd1* siRNA transfected, MGO-treated BMM when compared to non-transfected controls (Fig. 4T and U). Thus, defective thioredoxin but not glutathione production accounts for the dysregulation of bacterial control in MGO-treated BMM.

### 3.5 MGO irreversibly binds and inhibits the reductase activity of TXNRD1 and converts the enzyme into a NADPH oxidase

**Fig. 5:**
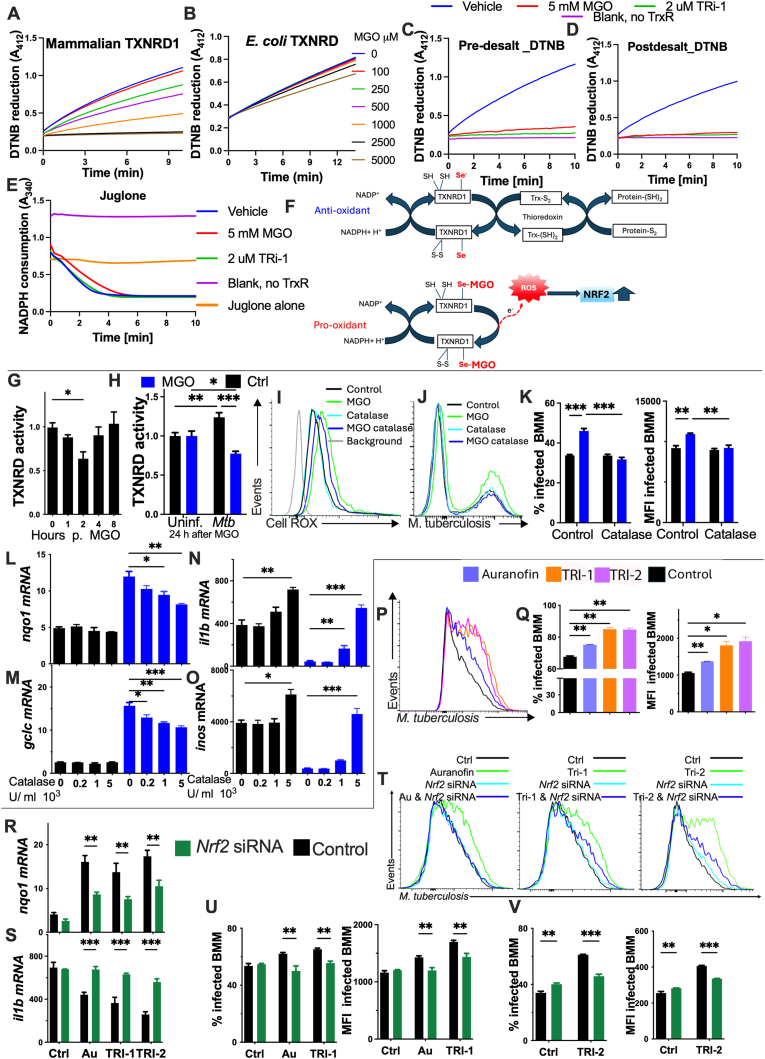
MGO inhibits the reductase activity of TXNRD1 and converts the enzyme into a NADPH oxidase **(A, B)** Inhibition of human TXNRD1 and *E. coli* TXNRD by MGO was followed by DTNB reduction as described in Methods section. **(C)** TXNRD1 250 nM was added to MGO or TRI-1 and incubated for 40 min with NADPH as described in Methods. Then, the mix was diluted 20 times and added to 0.25 mM NADPH and 2.5 mM DTNB. TNB formation was followed by reading A_412_. **(D and E)** The TXNRD1-compound mix was desalted using a Zeba 40k column to remove unbound compound, diluted 20 times and 0.25 mM NADPH and 2.5 mM DTNB were added to follow TNB formation at A_412_. The remaining desalted mix was added to 50 μM juglone and 0.25 mM NADPH and NADPH consumption was followed at A_340_. **(A**–**E)** The curves represent the mean of duplicates but the experiment was independently repeated two more times in singlets, showing the same results. **(F)** MGO inhibit the high reactive selenocysteine residue of TXNRD1 and convert the enzyme to pro-oxidant enzyme, producing ROS, contributing to the NRF2 responses in BMM treated with MGO. **(G and H)** The TXNRD activity in lysates of BMM was measured by DTNB reduction at different time points after incubation with MGO and in presence or absence of *Mtb*-infection. The TXNRD activity was normalized to the protein concentration of the lysates and the relative activity of each sample as compared to that of untreated/non-infected controls. **(I)** BMM were treated with MGO and/or with 10^3^ U/ml catalase. A representative histogram of Cell ROX labelling 24h after treatment is shown. **(J and K)** BMM were treated with MGO and/or with 10^3^ U/ml catalase and infected with *Mtb* 4 h after treatment. A representative histogram, the percentage of infected BMM and the *Mtb*-GFP MFI gated on infected cells 5 days after infection are shown. **(L**–**O)** BMM were treated with MGO and with the indicated concentrations of catalase and infected with *Mtb* 4h after treatment. The levels of *nqo1*, gclc, *il1b* and *inos* mRNA were determined by RT-PCR 24 h after infection. **(P and Q)** BMM were incubated with either 0.3 μM auranofin, 2 μM TRI-1, or 3 μM TRI-2 before infection with *Mtb-*GFP at a MOI of 3:1. A representative histogram, the percentage of infected BMM, and the GFP MFI gated on infected cells were determined 5 days after infection. **(R and S)** BMM were transfected with *Nrf2* and control siRNA, treated with either auranofin, TRI-1, and TRI-2 24 h after, and infected with *Mtb* 4 h after treatment. Total RNA was extracted 24 h after the infection and the relative concentration of *nqo1* and *il1b* mRNA were determined by real time PCR. **(T**–**V)** BMM were transfected with *Nrf2* and control siRNA, treated with either auranofin, TRI-1, and TRI-2 24h after and infected with *Mtb* 4 h after treatment. Representative histograms, the percentage of infected BMM and the *Mtb*-GFP MFI gated on infected cells 5 days after infection are shown. (The effect of TRI-2 was determined in independent experiments). **(G**–**V)** The mean ± SEM determinations of 3 independent BMM cultures per group are shown. **(H, K, R, S, U, V)** Differences are significant by 2-way ANOVA, **(G, L-O, Q)** 1-way ANOVA at ∗p ≤ 0.05, ∗∗p ≤ 0.01 and ∗∗∗p ≤ 0.001.

We then studied the molecular mechanisms by which TXNRD1 could mediate the MGO-induced NRF2 activation. First, whether MGO could directly bind to and regulate the enzymatic activity of recombinant mammalian TXNRD1 was studied using DTNB as a substrate and assessing NADPH consumption. Controls without TXNRD1, with and without MGO, were used to exclude any nonenzymatic reaction of MGO with DTNB or NADPH. MGO inhibited the reductase activity of TXNRD1 in a dose-dependent manner (Fig. 5A and Supplementary Fig. 4A). There was no evidence of MGO acting as a substrate since NADPH was not oxidized during incubation in the absence of DTNB (Supplementary Fig. 4B). NADPH was required for the reductase activity of TXNRD1 (Supplementary Fig. 4C). DTNB was not reduced, and NADPH was not oxidized when TXNRD1 was excluded from the reaction (Supplementary Fig. 4D and E). The preincubation of TXNRD1 with NADPH was required for MGO inhibition, suggesting that TXNRD1 needs to be pre-reduced to expose the selenolate of the selenocysteine in the enzyme, thereby allowing MGO inhibition (Supplementary Fig. 4F).

The selenocysteine active centre of mammalian TXNRD1 is not present in the prokaryotic orthologues [39]. MGO inhibited *E. coli* TXNRD only at high concentrations, suggesting the preference of MGO for the selenocysteine as it is a more efficient inhibitor of the mammalian enzyme (Fig. 5B). MGO did not act as a substrate of the bacterial TXNRD and DTNB was not reduced and NADPH was not consumed in absence of the bacterial TXNRD (Supplementary Fig. 4G–I). In line with this, MGO at the concentrations that impaired the intracellular control of *Mtb* by BMM did not alter the growth of *Mtb* in axenic cultures (Supplementary Fig. 4J).

The inhibition of TXNRD1 by electrophilic molecules may convert the enzyme into a selenium-compromised thioredoxin reductase-derived apoptotic protein (SecTRAP) that retain efficient NADPH oxidase activity, despite not being able to reduce their cognate substrate thioredoxin, thereby producing ROS [41,42]. The SecTRAP formation requires the irreversible inhibition of TXNRD1. TXNRD1 was incubated with the specific inhibitor TRI-1 as a positive control and MGO at concentrations sufficient to fully inhibit the C-terminal active site before desalting through an exclusion column to remove unbound compound (Fig. 5C). TXNRD1 inhibition by MGO was irreversible since the natural activity was not regained after desalting (Fig. 5D) but evidenced SecTRAP activity as assessed by the subsequent incubation of inhibited TXNRD1 with juglone (Fig. 5E). Juglone is a substrate which uses both the C- and N-terminal active site of TXNRD1, which means that TXNRD1 can reduce juglone but not DTNB (that depends on the C-terminal selenocysteine containing active site) (Fig. 5E and Supplementary Fig. 4K). Thus, MGO (like TRI-1) irreversibly inhibits TXNRD1 and converts it into a NADPH oxidase which correlated with the observation that TXNRD1 activity mediated NRF2 activation (Fig. 5F).

Incubation with MGO transiently diminished the TXNRD1 activity in BMM, while MGO inhibition of TXNRD1 activity was sustained in BMM infected with *Mtb* (Fig. 5G and H). The TXNRD1 activity of BMM incubated with CBR-470-1 was also reduced (Supplementary Fig. 4L).

ROS were generated by MGO-treatment of BMM or by *Mtb* infection and were reduced by addition of catalase that removes H_2_O_2_ from the cellular environment [43] (Fig. 5I and Supplementary Fig. 4M and N). Incubation with 10^3^ U/ml catalase restored the control of *Mtb* in MGO-treated BMM (Fig. 5J and K). The levels of antioxidant transcripts were decreased in *Mtb* MGO BMM, but not in untreated infected BMM, when treated with catalase (Fig. 5L and M). Instead, *il1b* and *inos* mRNA levels in *Mtb* MGO BMM increased after incubation with catalase (Fig. 5N and O), indicating that an increased oxidative stress could mediated the NRF2 activation, the impaired inflammatory responses and the bacterial control in *Mtb* MGO BMM.

To corroborate that inhibition of TXNRD1 in BMM accounts for the defective intracellular bacterial control, BMM were treated with TRI-1, TRI-2 and auranofin, potent TXNRD1 inhibitors [29,44]. The incubation with these inhibitors increased the *Mtb* levels in BMM at 5 days after infection (Fig. 5P and Q) and increased the accumulation of antioxidant genes in *Mtb-*infected and uninfected BMM (Fig. 5R and Supplementary Fig. 4O–Q). The expression of *il1b* mRNA and nitrite in supernatants were decreased in *Mtb* BMM treated with the TXNRD1 inhibitors as compared to non-treated controls (Fig. 5S and Supplementary Fig. 4R). *Mtb*-infected BMM treated with TXNRD1 inhibitors showed lower *nqo1* and higher *il1b* mRNA levels when transfected with *Nrf2* siRNA as compared to non-transfected controls (Fig. 5R and S), and *Mtb levels* in auranofin, TRI-1 and TRI-2 treated BMM diminished if transfected with *Nrf2* siRNA (Fig. 5T–V). In line with this, *Txnrd1* siRNA transfected BMM showed lower *nqo1* mRNA levels after incubation with the inhibitors and, as expected, decreased levels of *txnrd1* mRNA (Supplementary Fig. 4S and T).

Hence, the incubation of BMM with TXNRD1 inhibitors recapitulated results observed in MGO-treated BMM. This supports that MGO binds to TXNRD1 and hampers the intracellular control of *Mtb* growth in BMM in a NRF2 dependent manner.

When attempting to reproduce the results with *Nrf2* siRNA using *Nrf2*^*−/−*^ BMM, we observed that *Mtb-infected* or uninfected *Nrf2*^*−/−*^ BMM showed diminished survival after incubation with either MGO, auranofin, TRI-1 or TRI-2 at conditions in which WT BMM were highly viable (Supplementary Fig. 5A–C). Incubation with catalase protected *Nrf2*^*−/−*^ BMM from MGO toxicity (Supplementary Fig. 5D). The i.p. administration of 10 mg/kg auranofin was toxic for *Nrf2*^*−/−*^ mice even in absence of *Mtb* infection (Supplementary Fig. 5E). The residual *nrf2* levels after silencing probably accounts for the survival of *Nrf2* siRNA BMM treated with MGO or the thioredoxin reductase inhibitors.

Lipid peroxidation, or the oxidative degradation of lipids, results in oxidative stress and cell damage, is controlled by NRF2-regulated molecules. Lipid peroxidation is the core reaction of ferroptosis, a necrotic cell death mechanism considered to be host detrimental during *Mtb* infection [45,46]. We found that, with a peak response at 1 h after MGO incubation (Supplementary Fig. 5F), MDA levels in BMM augmented when incubated with increasing concentrations of MGO (Supplementary Fig. 5G). Macrophage infection with *Mtb-*induced lipid peroxidation levels that were synergically increased if BMM were preincubated with MGO before the infection (Supplementary Fig. 5H). MDA levels were increased in *Nrf2*^−/−^ BMM as compared to those in WT when incubated with either MGO or auranofin (Supplementary Fig. 5I and J). *Mtb*-infected *Nrf2*^*−/−*^ BMM treated or not with MGO showed higher MDA levels than the WT controls (Supplementary Fig. 5K). However, incubation with the iron chelator deferoxamine (DFO) which hampers ferroptosis, did not prevent the death of *Nrf2*^*−/−*^ BMM treated with MGO, auranofin, TRI-1 or TRI-2 (Supplementary Fig. 5L). The addition of the ferroptosis inducer RSL3 resulted in a marker reduced of viability of WT BMM. Incubation with 100 μM DFO or ferrostatin-1 (a lipid peroxidation inhibitor) both prevented BMM death by RSL3, controlling the efficiency of DFO in impairing ferroptosis (Supplementary Fig. 5M).

Thus, NRF2 activation prevents BMM from ROS-mediated, non ferroptotic death.

### 3.6 Interferon-γ abrogates the suppression of protective macrophage responses triggered by MGO

**Fig. 6:**
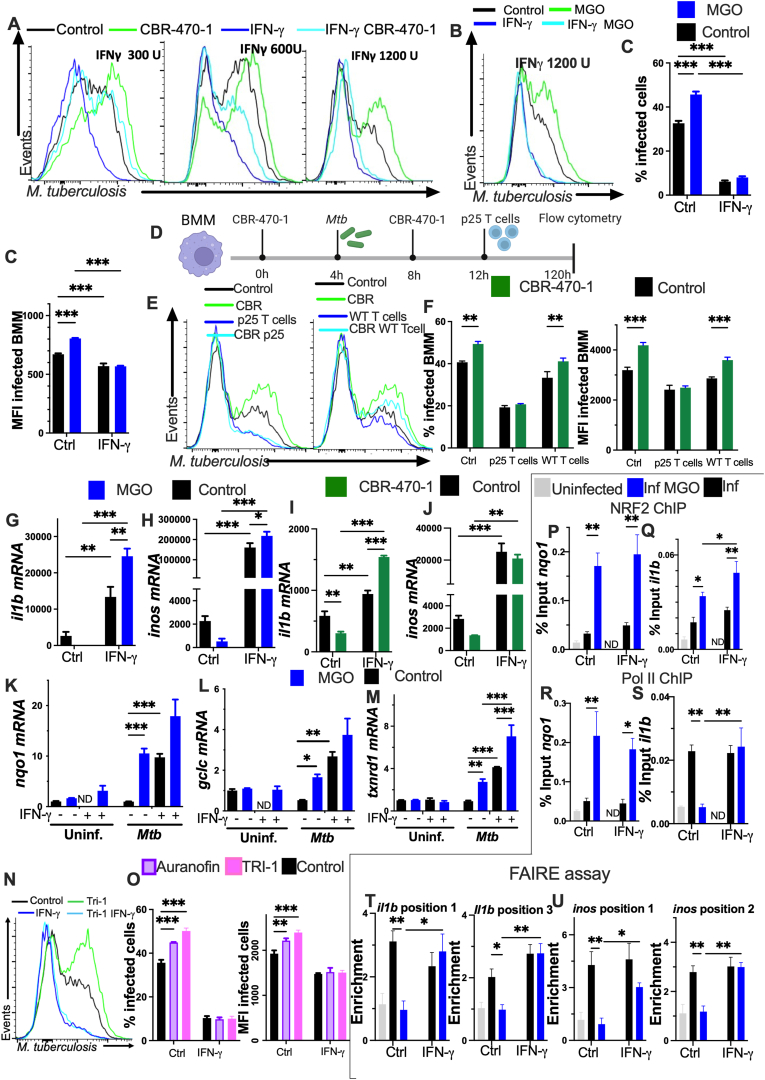
Interferon-γ abrogates the MGO suppression of protective macrophage responses. **(A)** BMM were incubated with recombinant IFN-γ at the indicated concentrations, treated with CBR-470-1 48 h later and infected with *Mtb-*GFP 4 h after the treatment. IFN-γ was replenished 4 h after infection. Representative histograms of *Mtb*-GFP-labelled BMM 5 days after infection are shown. **(B and C)** BMM were cultured with 1200 U/ml IFN-γ, incubated with MGO 48 h after, infected with *Mtb-*GFP 4 h after treatment and IFN-γ was replenished 4 h after infection. A representative histogram, the percentage of infected BMM and the MFI gated on infected BMM 5 days after infection are displayed. **(D)** BMM were treated with CBR-470-1, 4 h after treatment were infected with *Mtb* and CBR-470-1 was replenished 4h after the infection. BMM were co-cultured with p25 transgenic or wild type (WT) CD4 T cells. **(E and F)** Representative histograms, the percentage of infected BMM and the *Mtb*-GFP MFI gated on infected cells 5 days after infection are shown. **(E)** Panels are within the same experiment. **(G**–**M)** BMM were incubated with IFN-γ, treated with either MGO or CBR.470-1 48 h later and infected with *Mtb* 4 h after treatment. Total RNA was isolated 24 h after infection and the *il1b*, *inos, nqo1*, *gclc* and *txnrd1* mRNA levels were determined by RT-PCR. **(N and O)** BMM were incubated with IFN-γ, treated with either TRI-1 or auranofin 48h after IFN-γ stimulation and infected with *Mtb* 4 h after treatment. Representative histogram (TRI-1 only), the percentage of infected BMM and the *Mtb*-GFP MFI 5 days after infection are depicted. **(P–S)** BMM treated for 4 h with MGO, infected then with *M. bovis* BCG. 4 h thereafter binding of either NRF2 **(P and Q)** or RNA Polymerase II **(R and S)** to *nqo1* and *il1b* genes were measured in BMM lysates by ChIP-qPCR. The % input is depicted in comparison to non-immunoprecipitated chromatin. **(T and U)** BMM treated for 4 h with MGO, infected then with *M. bovis* BCG. 4 h after the quantification of open chromatin at positions 1 and 3 in *il1b*, and positions 1 and 2 in the *inos* sequences was evaluated using the FAIRE assay (see Supplementary Fig. 5O for genomic localization and features of the analysed regions). **(C, F, G-M, O, Q, S, U)** Data are mean ± SEM from *n* = 3 independent samples per group. Differences are significant at ∗p ≤ 0.05, ∗∗p ≤ 0.01 and ∗∗∗p ≤ 0.001, 2-way ANOVA.

IFN-γ enhances the mycobactericidal responses of macrophages and is non-redundant for the control of *Mtb* infection [47,48]. The effect of MGO in the control of *Mtb* infection by IFN-γ activated BMM was then studied. The IFN-γ stimulation of BMM before and during infection diminished the intracellular *Mtb* levels (Fig. 6A–C and Supplementary Fig. 6A–C). The bacterial levels in CBR-470-1 treated, *Mtb*-infected BMM when incubated with 300 or 600 U/ml IFN-γ were lower than those in CBR-470-1 treated BMM not activated with IFN-γ, but higher than those in IFN-γ-stimulated CBR-470-1 untreated BMM (Fig. 6A and Supplementary Fig. 6A and B). Instead, incubation of BMM with 1200 U/ml IFN-γ abrogated the ability of MGO or CBR-470-1 to suppress proper bacterial control (Fig. 6A–C and Supplementary Fig. 6C). The uptake of *Mtb* by IFN-γ-stimulated or untreated BMM was similar (Supplementary Fig. 6D).

The *Mtb levels* in IFN-γ-treated *Keap1* siRNA or control siRNA transfected BMM were similar and significantly lower than those from BMM not treated with IFN-γ (Supplementary Fig. 6E and F).

BMM incubated with IFN-γ only once, before the infection with *Mtb* showed similar bacterial levels regardless these were treated or not with MGO (Supplementary Fig. 6G and H).

Mycobacteria-specific T cells release IFN-γ when activated by infected macrophages. Since, only high concentrations of IFN-γ reverted the deficient control of *Mtb* in BMM treated with MGO or CBR-470-1, we tested if the coculture of BMM with mycobacteria-specific T cells abrogated the suppression of *Mtb* control (Fig. 6D). The co-culture of BMM with syngeneic CD4 T-cell receptor transgenic mycobacteria-specific p25 but not with WT CD4 T cells lowered the intracellular *Mtb* to similar levels in BMM incubated or not with CBR-470.1 (Fig. 6E and F). The co-culture of p25T cells with *Mtb* infected allogeneic H2^d^ BMM did not decrease bacterial levels confirming that cognate T cell recognition was required for the control of the intracellular BMM infection (Supplementary Fig. 6I).

The levels of *inos* and *il1b* mRNA in either MGO-, CBR-treated or untreated IFN-γ stimulated, *Mtb i*nfected *BMM* were markedly increased as compared to those in IFN-γ untreated BMM (Fig. 6G–J), and titers of antioxidant transcripts in CBR-470-1 or MGO-treated BMM were not impaired by the incubation with IFN-γ (Fig. 6K–M and Supplementary Fig. 6J–L). IFN-γ also restored the control of *Mtb* intracellular growth in BMM incubated with TXNRD1 inhibitors (Fig. 6N and O).

Using Chip-PCR we observed NRF2 enrichment at previously described binding sites [31] in the proximity of *il1b* and *nqo1* genes in MGO-treated infected BMM, even after IFN-γ stimulation, but not in controls (Fig. 6P and Q and Supplementary Fig. 6M).

Binding of RNA polymerase II (Pol II) (indicative of mRNA transcription) to *nqo1* was enriched in MGO treated BMM, incubated or not with IFN-γ (Fig. 6R). Instead, the binding of Pol II to *il1b* and *inos* genes was hampered in MGO-treated mycobacteria (BCG)-infected BMM (Fig. 6S and Supplementary Fig. 6N). The stimulation of BMM with IFN-γ allowed Pol II binding even in presence of MGO, consistent with the mRNA levels of the specific mRNA measured (Fig. 6S and Supplementary Fig. 6N).

Using the FAIRE (formaldehyde-assisted isolation of regulatory elements) assay [49], chromatin accessibility to regulatory regions proximal and distal to the transcription start sites of *il1b* and *inos* genes were found to be increased in infected BMM (Fig. 6T and U). The regions studied showed positive histone acetylation and methylation marks, and binding to different transcription factors but not necessarily to NRF2 (Supplementary Fig. 6O), suggesting that the NRF2-mediated inhibition of *inos* and *il1b* genes was at least in part independent on the NRF2 binding to these positions. The treatment of infected BMM with MGO reduced the chromatin permissiveness to some of these positions (Fig. 6T and U). The stimulation with IFN-γ of MGO-treated and infected BMM recovered chromatin accessibility in these positions to similar levels than those measured in infected BMM (Fig. 6T and U). The chromatin in the promoter of *nqo1* showed increased accessibility after MGO stimulation, which was unaltered after incubation of BMM with IFN-γ, suggesting differential responses by diverse genes to MGO and/or IFN-γ (Supplementary Fig. 6P), while chromatin accessibility to the cd3g promoter after either mycobacterial infection, MGO treatment and/or stimulation with IFN-γ were similar. Our data supports a role of IFN-γ in abolishing the MGO effects as the result of the regulation of immune protective genes at the epigenetic level.

Altogether, IFN-γ revokes the suppressive effect of MGO, CBR-470-1 and of the inhibitors of the TXNRD1 on the intracellular growth control of *Mtb*, without impairing the expression of NRF2 downstream genes.

### 3.7 MGO and auranofin diminish the control of *M. tuberculosis* infection in mice

**Fig. 7:**
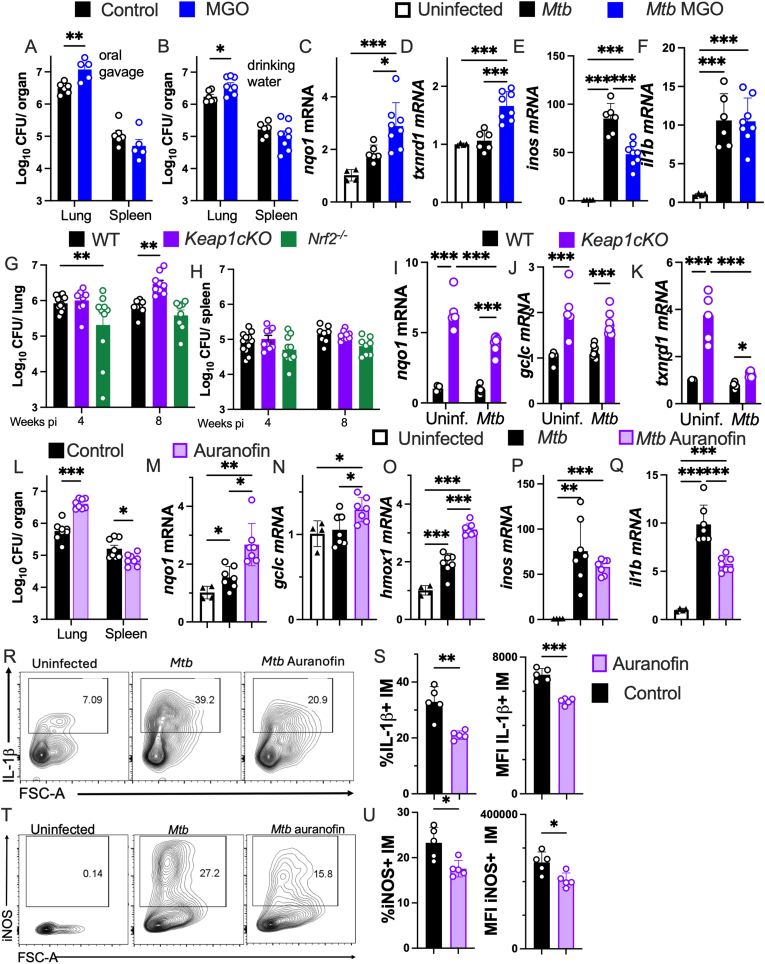
Administration of MGO or auranofin increased NRF2 downstream responses and impaired the control of *M. tuberculosis* infection in mice **(A)** Mice (C57BL/6) were infected with 200 CFU via aerosol route, treated with 200 μl of 500 mg/kg MGO (dissolved in water) by oral gavage starting 2 weeks after infection for 6 days, and sacrificed 3 weeks after infection. **(B)** Other group of mice were administered with 1 % MGO in drinking water for 10 weeks before infection with *Mtb.* Treatment with MGO continued for 4 weeks after infection when sacrificed. The log_10_ CFU in lungs and spleens are displayed. **(C–F)** Total RNA was extracted from lungs of mice treated or not with MGO in drinking water at 4 weeks after *Mtb* infection. The individual and the mean fold increase of *nqo1*, *txnrd1*, *inos* and *il1b* mRNA levels in lungs as compared to lungs from untreated/uninfected controls are depicted. **(G and H)** WT, *Keap1 cKO* and *Nrf2*^*−/−*^ mice were infected with *Mtb* and sacrificed 4 and 8 weeks after infection. The log_10_ CFU in lungs and spleens of mice are depicted**.** **(I**–**K)** Total RNA was extracted from lungs of *Keap1cKO* and WT mice 4 weeks after infection with *Mtb* and from uninfected WT controls. The *nqo1*, gclc and *txnrd1* mRNA levels in lungs are shown. **(L)** Mice were treated with 10 mg/kg auranofin in PBS daily (5 days/week) during 4 weeks starting 1 day after *Mtb* infection and sacrificed a day after the last dose of auranofin. The log_10_ CFU in lungs and spleens of treated and control mice are illustrated. **(M**–**Q)** The *nqo1, gclc, hmox1*, *inos* and *il1b* mRNA levels in lungs from uninfected, infected, and auranofin treated and infected mice are shown. **(R–U)** Representative contour plots, the frequency and the MFI of IL-1β+ and iNOS+ (IM) gated as SiglecF-, CD11b+ and F4/80+ in the lungs of mice treated or not with auranofin one month after *Mtb* infection. **(A-Q, T-W)** The individual and mean values ± SEM are shown. (A, B, G, H, L, T-W) Differences are significant by unpaired Student's *t*-test with Welch correction, (C–F,G,H,M-Q) one-way ANOVA, and (I–K) two-way ANOVA at ∗p ≤ 0.05, ∗∗p ≤ 0.01 and ∗∗∗p ≤ 0.001.

Whether MGO and auranofin administration increased the susceptibility of mice to the aerosol infection with *Mtb* was then analysed. Mice administered with MGO either by oral gavage or in drinking water [50,51] showed increased *Mtb* titers in lungs but not spleens as compared with untreated controls (Fig. 7A and B). Lungs of mice treated with MGO showed increased titters of antioxidant transcripts (Fig. 7C and D), while levels of *inos* (but not *il1b*) mRNA were lower than those from controls (Fig. 7E and F).

The outcome of infection with *Mtb* of *Keap1* *cKO* and *Nrf2*^*−/−*^ mice was then evaluated. Lungs from *Keap1* *cKO* mice showed increased bacterial levels at 8 (but not 4 weeks) after infection, whereas *Nrf2*^*−/−*^ mice showed a moderate reduction of lung CFU at 4 weeks after infection. *Mtb* titters in the spleens of WT and mutant mice were similar (Fig. 7G and H). Lungs from *Keap1* *cKO* mice showed increased accumulation of antioxidant transcripts when measured 4 weeks after *Mtb* infection (Fig. 7I–K).

Mice administered with 10mg/kg auranofin i.p. daily during infection showed significantly increased *Mtb* levels in lungs but not spleens as compared to controls without losing weight, suggesting auranofin at the doses administered was not toxic (Fig. 7L and Supplementary Fig. 7A and B). The differential effect of MGO and auranofin in lungs and spleens, may relate to diverse organ-specific responses to these molecules, or that the systemic dissemination of the infection from the lung is not modulated by MGO and auranofin. Increased levels of NRF2-regulated transcripts were present in lungs from auranofin treated mice as compared to those of controls (Fig. 7M − O and Supplementary Fig. 7C and D). Lower levels of *il1b* (but not *inos*) mRNA were determined in the lung from auranofin treated *Mtb* infected mice than in those from controls (Fig. 7P and Q).

The infection with *Mtb* led to an enrichment of inflammatory macrophages (IM) in the lung, while the percentage of alveolar macrophages (AM) decreased (Supplementary Fig. 7E). The density of IM and AM in auranofin treated and untreated *Mtb* infected mice was similar (Supplementary Fig. 7E). Pulmonary IM expressed high IL-1β and iNOS levels during *Mtb* infection (Fig. 7R and S and Supplementary Fig. 6F). The expression of iNOS and IL-1β producing pulmonary IM from auranofin treated mice was decreased as compared to that of infected controls (Fig. 7R–U). IM co-expressed iNOS and IL-1β and showed high expression of CD11b, MHCII and CD86 activation markers (Supplementary Fig. 7G). The IL-1β and iNOS levels produced by AM were also increased after infection with *Mtb* but decreased in the lungs of auranofin treated mice as compared to controls (Supplementary Fig. 7H–J).

Thus, treatment of mice with MGO and auranofin increased levels of NRF2 downstream genes, diminished levels of protective immune responses in pulmonary IM and increased the susceptibility of mice to the infection with *Mtb*.

## 4 Discussion

The dual burden of TB and DM has attracted much attention in the past decade as DM prevalence has increased dramatically in countries already afflicted with a high burden of TB [52]. Macrophages from rodents or TB patients with DM have a lowered activation state compared with those from non-diabetic controls [[53], [54], [55], [56]].

Hyperglycemia-dependent mitochondrial overproduction of superoxide has been shown to alter glucose catabolism resulting in increased glycolytic intermediates such as MGO [57].

Here we showed that exogenous addition or endogenous production of MGO mediates a TXNRD1 and NRF2-dependent impairment in *Mtb* control in macrophages. While the NRF2 system protected against oxidative stress in MGO-treated BMM, as also shown by others [58], a consequence of NRF2 activation is the inhibition of immune/inflammatory responses [31]. MGO-induced the NRF2 mediated inhibition of IL1β expression that accounted for the deficient handling of the intracellular bacterial levels by macrophages. Moreover, the NRF2 overexpression was not only required but was sufficient for impairing *Mtb* control in macrophages. In support for this mechanism underlying *Mtb* susceptibility in DM, we observed weakened control of *Mtb,* increased NRF2 and diminished levels of inflammatory transcripts in BMM incubated with high glucose levels and in MGO-treated human macrophages.

Electrophilic compounds that activate NRF2 by targeting Keap1 may also inhibit TXNRD1 [40]. In turn, TXNRD1 has been proposed as a potent regulator of NRF2 [40]. In agreement with our results, prior studies have shown that MGO impaired the TXNRD1/thioredoxin system [59,60]. TXNRD1 mediated NRF2 activation, the inhibition of inflammatory responses and the defective control of *Mtb* infection in BMM incubated with MGO or CBR-470-1. Instead, glutathione is involved in the detoxification of MGO by the glyoxalase system [61], and it is likely that the impairment of GSH formation by BSO stabilized MGO, resulting in the increased intracellular bacterial levels observed.

A direct targeting of Keap1 by MGO [18,62,63], although not excluded by our data, proved not to be sufficient to drive NRF2 activation upon TXNRD1 deficiency.

While MGO readily reacts with lipids, DNA and cellular and extracellular proteins [12], the modulation of the macrophage responses was mediated by the MGO inhibition of TXNRD1. As also shown for other reactive molecules [64], MGO did not only inhibit the reductase but also transformed the protein to a pro-oxidant enzyme with NADPH oxidase activity. When incubated with MGO, TXNRD1 catalysed the efficient reduction of juglone in a reaction which, in contrast to reduction of most other substrates of TXNRD1, is not dependent upon an intact selenocysteine residue of the enzyme, correlating with the prooxidant activity of the SecTRAP [65]. MGO has been shown to bind to lysine and arginine and also reversibly to cysteine [66]. The preference of MGO for selenocysteine is suggested by the inhibition of the mammalian but not the bacterial TXNRD (that lacks selenocysteine) and characterized by an irreversible binding to the enzyme. The SecTRAP allow NRF2 activation, as also showed in previous studies using TXNRD1 inhibitors [67,68]. We show that MGO impaired NO formation but increased endogenous ROS formation as well as lipid peroxidation, confirming previous studies [69]. The depletion of ROS by catalase in MGO treated BMM further impaired NRF2 activation, increased the levels of inflammatory transcripts and improved *Mtb* control. On the other hand, NRF2 prevented MGO mediated lipid peroxidation and BMM death.

Since MGO can bind to multiple targets, TRI-1, TRI-2 and auranofin inhibitors of TXNRD1 were used. Incubation with these molecules decreased protective inflammatory responses that impaired the *Mtb* control in BMM in a NRF2-dependent manner. In agreement with this, auranofin mediated TXNRD1 inhibition has been shown to inhibit IL-1β production [70,71]. These compounds also induced NRF2-regulated responses and inhibited immune transcripts in human macrophages. Auranofin administration increased the susceptibility of mice to infection with *Mtb* and impaired IL-1β and iNOS expression in pulmonary macrophages *in vivo*.

IFN-γ secreted by T cells is required for macrophages to attain a mycobactericidal state [72]. IFN-γ restored protective immune response and the control of *Mtb* infection in MGO- or CBR-470-1 treated BMM, without hampering the binding of NRF2 to *il1b* promoter and the expression of NRF2 downstream genes. The coculture of infected BMM with mycobacterial specific T cells which produce IFN-γ [73], also reverted the MGO- suppression of *Mtb* growth control in MGO-treated BMM. One important function of IFN-γ is the reversal of endotoxin tolerance and restoration of inflammatory cytokine production, which has been shown *in vitro* and *in vivo* in mice and humans [[74], [75], [76]]. We propose that MGO induces NRF2 binding to regulatory elements and impairs chromatin accessibility in regulatory positions in the *il1b* and *inos* genes that bind NRF2 or not. IFN-γ did not impede NRF2 binding but restored chromatin accessibility to these regions in MGO-treated BMM. In line with this, IFN-γ has been known to prevent and potentially reverse LPS tolerance via epigenetic mechanisms [75,77,78]. While molecular mechanisms behind IFN-γ -mediated reconstitution of macrophage responses remain to be explored, our data suggests that the resistance to TB in DM patients may be improved by immunization or vaccination. This study also supports the use of scavengers of reactive dicarbonyl species, or antioxidants such as N-Acetyl cysteine (NAC), cysteine, glutathione, which are used or proposed for treatment of different morbidities as candidates for developing treatment of TB in DM patients.

Altogether, we propose and identify the mechanisms by which alterations of carbohydrate metabolism in DM may hinder the control of *Mtb* infection.

## CRediT authorship contribution statement

**Hanxiong Li:** Writing – review & editing, Investigation, Formal analysis, Data curation, Conceptualization. **Ruining Liu:** Writing – review & editing, Investigation, Formal analysis. **Gokul Raj Kathamuthu:** Writing – review & editing, Investigation, Formal analysis. **Radosveta Gencheva:** Writing – review & editing, Investigation, Formal analysis. **Zhen Gong:** Investigation, Methodology, Writing – review & editing. **Axel Tobias Scholz:** Investigation. **Mohammad Alzrigat:** Formal analysis, Data curation. **Lucia Coppo:** Writing – review & editing, Investigation, Formal analysis, Data curation, Conceptualization. **Elias S.J. Arnér:** Writing – review & editing, Resources, Formal analysis, Conceptualization. **Martin E. Rottenberg:** Writing – review & editing, Writing – original draft, Resources, Funding acquisition, Formal analysis, Data curation, Conceptualization.

## Data availability

The raw and processed RNA sequencing data have been deposited in the publicly accessible Gene Expression Ommnibus (GEO) NIH database repository and can be accessed using GEO accession number GSE271061. The authors declare that data supporting the findings of this study are available within the paper and its supplementary information files. Source data (individual FACS plots, or detailed gating strategies) are available on request from the corresponding author.

## Funding information

This study was supported by the Swedish Heart and Lung foundation 2021-23/20200697, the 10.13039/501100004359Swedish Research Council 2019-01691 and 2019–04725, the 10.13039/501100001728Swedish Institute for Internationalization of Research and Higher Education (STINT) 4–1796/2014, the 10.13039/501100002794Chinese Scholarship Council and the Karolinska Institutet to MR. Support by the Swedish Cancer Society (21 1463 Pj), The 10.13039/501100004359Swedish Research Council (2021–02214), The 10.13039/100020651Cayman Biomedical Research Institute (CABRI), and The Hungarian 10.13039/501100018818National Research, Development and Innovation Office (10.13039/501100011019NKFIH),under the National Laboratories Program (National Tumor Biology Laboratory (2022-2.1.1-NL-2022-00010)), the Hungarian Thematic Excellence Program (under project TKP2021-EGA-44), and Project Grant K 146277 was granted to EA.

## Declaration of competing interest

None.

## Data Availability

The raw and processed RNA sequencing data have been deposited in the publicly accessible Gene Expression Ommnibus (GEO) NIH database repository and can be accessed using GEO GSE271061.

## References

[1] WHO (2016).

[2] Jeon C.Y., Murray M.B. (2008). Diabetes mellitus increases the risk of active tuberculosis: a systematic review of 13 observational studies. PLoS Med..

[3] Dooley K.E., Chaisson R.E. (2009). Tuberculosis and diabetes mellitus: convergence of two epidemics. Lancet Infect. Dis..

[4] Lonnroth K., Castro K.G., Chakaya J.M., Chauhan L.S., Floyd K., Glaziou P., Raviglione M.C. (2010). Tuberculosis control and elimination 2010-50: cure, care, and social development. Lancet.

[5] Cohen S.B., Gern B.H., Delahaye J.L., Adams K.N., Plumlee C.R., Winkler J.K., Sherman D.R., Gerner M.Y., Urdahl K.B. (2018). Alveolar macrophages provide an early Mycobacterium tuberculosis niche and initiate dissemination. Cell Host Microbe.

[6] Suzuki T., Yamamoto M. (2015). Molecular basis of the Keap1-Nrf2 system. Free Radic. Biol. Med..

[7] Tebay L.E., Robertson H., Durant S.T., Vitale S.R., Penning T.M., Dinkova-Kostova A.T., Hayes J.D. (2015). Mechanisms of activation of the transcription factor Nrf2 by redox stressors, nutrient cues, and energy status and the pathways through which it attenuates degenerative disease. Free Radic. Biol. Med..

[8] Muri J., Kopf M. (2021). Redox regulation of immunometabolism. Nat. Rev. Immunol..

[9] Shah M.S., Brownlee M. (2016). Molecular and cellular mechanisms of cardiovascular disorders in diabetes. Circ. Res..

[10] Ramasamy R., Yan S.F., Schmidt A.M. (2006). Methylglyoxal comes of AGE. Cell.

[11] Hanssen N.M.J., Teraa M., Scheijen J., Van de Waarenburg M., Gremmels H., Stehouwer C.D.A., Verhaar M.C., Schalkwijk C.G. (2021). Plasma methylglyoxal levels are associated with amputations and mortality in severe limb ischemia patients with and without diabetes. Diabetes Care.

[12] Schalkwijk C.G., Methylglyoxal C.D.A. Stehouwer (2020). A highly reactive dicarbonyl compound, in diabetes, its vascular complications, and other age-related diseases. Physiol. Rev..

[13] Nowotny K., Jung T., Hohn A., Weber D., Grune T. (2015). Advanced glycation end products and oxidative stress in type 2 diabetes mellitus. Biomolecules.

[14] Moraru A., Wiederstein J., Pfaff D., Fleming T., Miller A.K., Nawroth P., Teleman A.A. (2018). Elevated levels of the reactive metabolite methylglyoxal recapitulate progression of type 2 diabetes. Cell Metab..

[15] Guo Q., Mori T., Jiang Y., Hu C., Osaki Y., Yoneki Y., Sun Y., Hosoya T., Kawamata A., Ogawa S., Nakayama M., Miyata T., Ito S. (2009). Methylglyoxal contributes to the development of insulin resistance and salt sensitivity in sprague-dawley rats. J. Hypertens..

[16] Brouwers O., Niessen P.M., Ferreira I., Miyata T., Scheffer P.G., Teerlink T., Schrauwen P., Brownlee M., Stehouwer C.D., Schalkwijk C.G. (2011). Overexpression of glyoxalase-I reduces hyperglycemia-induced levels of advanced glycation end products and oxidative stress in diabetic rats. J. Biol. Chem..

[17] Twarda-Clapa A., Olczak A., Bialkowska A.M., Koziolkiewicz M. (2022). Advanced glycation end-products (AGEs): formation, chemistry, classification, receptors, and diseases related to AGEs. Cells.

[18] Bollong M.J., Lee G., Coukos J.S., Yun H., Zambaldo C., Chang J.W., Chin E.N., Ahmad I., Chatterjee A.K., Lairson L.L., Schultz P.G., Moellering R.E. (2018). A metabolite-derived protein modification integrates glycolysis with KEAP1-NRF2 signalling. Nature.

[19] Rothchild A.C., Olson G.S., Nemeth J., Amon L.M., Mai D., Gold E.S., Diercks A.H., Aderem A. (2019). Alveolar macrophages generate a noncanonical NRF2-driven transcriptional response to Mycobacterium tuberculosis in vivo. Sci Immunol.

[20] Itoh K., Chiba T., Takahashi S., Ishii T., Igarashi K., Katoh Y., Oyake T., Hayashi N., Satoh K., Hatayama I., Yamamoto M., Nabeshima Y. (1997). An Nrf2/small maf heterodimer mediates the induction of phase II detoxifying enzyme genes through antioxidant response elements. Biochem. Biophys. Res. Commun..

[21] Okawa H., Motohashi H., Kobayashi A., Aburatani H., Kensler T.W., Yamamoto M. (2006). Hepatocyte-specific deletion of the keap1 gene activates Nrf2 and confers potent resistance against acute drug toxicity. Biochem. Biophys. Res. Commun..

[22] Clausen B.E., Burkhardt C., Reith W., Renkawitz R., Forster I. (1999). Conditional gene targeting in macrophages and granulocytes using LysMcre mice. Transgenic Res..

[23] Ziros P.G., Renaud C.O., Chartoumpekis D.V., Bongiovanni M., Habeos I.G., Liao X.H., Refetoff S., Kopp P.A., Brix K., Sykiotis G.P. (2021). Mice hypomorphic for Keap1, a negative regulator of the Nrf2 antioxidant response, show age-dependent diffuse goiter with elevated thyrotropin levels. Thyroid.

[24] Rothfuchs A.G., Gigliotti D., Palmblad K., Andersson U., Wigzell H., Rottenberg M.E. (2001). IFN-Alpha beta-dependent, IFN-Gamma secretion by bone marrow-derived macrophages controls an intracellular bacterial infection. J. Immunol..

[25] Rao Muvva J., Parasa V.R., Lerm M., Svensson M., Brighenti S. (2019). Polarization of human monocyte-derived cells with vitamin D promotes control of Mycobacterium tuberculosis infection. Front. Immunol..

[26] Mills E.L., Ryan D.G., Prag H.A., Dikovskaya D., Menon D., Zaslona Z., Jedrychowski M.P., Costa A.S.H., Higgins M., Hams E., Szpyt J., Runtsch M.C., King M.S., McGouran J.F., Fischer R., Kessler B.M., McGettrick A.F., Hughes M.M., Carroll R.G., Booty L.M., Knatko E.V., Meakin P.J., Ashford M.L.J., Modis L.K., Brunori G., Sevin D.C., Fallon P.G., Caldwell S.T., Kunji E.R.S., Chouchani E.T., Frezza C., Dinkova-Kostova A.T., Hartley R.C., Murphy M.P., O'Neill L.A. (2018). Itaconate is an anti-inflammatory metabolite that activates Nrf2 via alkylation of KEAP1. Nature.

[27] Carow B., Ye X., Gavier-Widen D., Bhuju S., Oehlmann W., Singh M., Skold M., Ignatowicz L., Yoshimura A., Wigzell H., Rottenberg M.E. (2011). Silencing suppressor of cytokine signaling-1 (SOCS1) in macrophages improves Mycobacterium tuberculosis control in an interferon-gamma (IFN-gamma)-dependent manner. J. Biol. Chem..

[28] Verdon C.P., Burton B.A., Prior R.L. (1995). Sample pretreatment with nitrate reductase and glucose-6-phosphate dehydrogenase quantitatively reduces nitrate while avoiding interference by NADP+ when the Griess reaction is used to assay for nitrite. Anal. Biochem..

[29] Stafford W.C., Peng X., Olofsson M.H., Zhang X., Luci D.K., Lu L., Cheng Q., Tresaugues L., Dexheimer T.S., Coussens N.P., Augsten M., Ahlzen H.M., Orwar O., Ostman A., Stone-Elander S., Maloney D.J., Jadhav A., Simeonov A., Linder S., Arner E.S.J. (2018). Irreversible inhibition of cytosolic thioredoxin reductase 1 as a mechanistic basis for anticancer therapy. Sci. Transl. Med..

[30] Rahman I., Kode A., Biswas S.K. (2006). Assay for quantitative determination of glutathione and glutathione disulfide levels using enzymatic recycling method. Nat. Protoc..

[31] Kobayashi E.H., Suzuki T., Funayama R., Nagashima T., Hayashi M., Sekine H., Tanaka N., Moriguchi T., Motohashi H., Nakayama K., Yamamoto M. (2016). Nrf2 suppresses macrophage inflammatory response by blocking proinflammatory cytokine transcription. Nat. Commun..

[32] Simon J.M., Giresi P.G., Davis I.J., Lieb J.D. (2012). Using formaldehyde-assisted isolation of regulatory elements (FAIRE) to isolate active regulatory DNA. Nat. Protoc..

[33] Teran G., Li H., Catrina S.B., Liu R., Brighenti S., Zheng X., Grunler J., Nylen S., Carow B., Rottenberg M.E. (2022). High glucose and carbonyl stress impair HIF-1-Regulated responses and the control of Mycobacterium tuberculosis in macrophages. mBio.

[34] MacMicking J.D., North R.J., LaCourse R., Mudgett J.S., Shah S.K., Nathan C.F. (1997). Identification of nitric oxide synthase as a protective locus against tuberculosis. Proc. Natl. Acad. Sci. U. S. A..

[35] Fremond C.M., Togbe D., Doz E., Rose S., Vasseur V., Maillet I., Jacobs M., Ryffel B., Quesniaux V.F. (2007). IL-1 receptor-mediated signal is an essential component of MyD88-dependent innate response to Mycobacterium tuberculosis infection. J. Immunol..

[36] Singh A., Venkannagari S., Oh K.H., Zhang Y.Q., Rohde J.M., Liu L., Nimmagadda S., Sudini K., Brimacombe K.R., Gajghate S., Ma J., Wang A., Xu X., Shahane S.A., Xia M., Woo J., Mensah G.A., Wang Z., Ferrer M., Gabrielson E., Li Z., Rastinejad F., Shen M., Boxer M.B., Biswal S. (2016). Small molecule inhibitor of NRF2 selectively intervenes therapeutic resistance in KEAP1-Deficient NSCLC tumors. ACS Chem. Biol..

[37] Franco R., Schoneveld O.J., Pappa A., Panayiotidis M.I. (2007). The central role of glutathione in the pathophysiology of human diseases. Arch. Physiol. Biochem..

[38] Drew R., Miners J.O. (1984). The effects of buthionine sulphoximine (BSO) on glutathione depletion and xenobiotic biotransformation. Biochem. Pharmacol..

[39] Lu J., Holmgren A. (2014). The thioredoxin antioxidant system. Free Radic. Biol. Med..

[40] Cebula M., Schmidt E.E., Arner E.S. (2015). TrxR1 as a potent regulator of the Nrf2-Keap1 response system. Antioxidants Redox Signal..

[41] Gencheva R., Arner E.S.J. (2022). Thioredoxin reductase inhibition for cancer therapy. Annu. Rev. Pharmacol. Toxicol..

[42] Cheng Q., Antholine W.E., Myers J.M., Kalyanaraman B., Arner E.S., Myers C.R. (2010). The selenium-independent inherent pro-oxidant NADPH oxidase activity of mammalian thioredoxin reductase and its selenium-dependent direct peroxidase activities. J. Biol. Chem..

[43] Preston T.J., Muller W.J., Singh G. (2001). Scavenging of extracellular H2O2 by catalase inhibits the proliferation of HER-2/Neu-transformed rat-1 fibroblasts through the induction of a stress response. J. Biol. Chem..

[44] Sabatier P., Beusch C.M., Gencheva R., Cheng Q., Zubarev R., Arner E.S.J. (2021). Comprehensive chemical proteomics analyses reveal that the new TRi-1 and TRi-2 compounds are more specific thioredoxin reductase 1 inhibitors than auranofin. Redox Biol..

[45] Amaral E.P., Costa D.L., Namasivayam S., Riteau N., Kamenyeva O., Mittereder L., Mayer-Barber K.D., Andrade B.B., Sher A. (2019). A major role for ferroptosis in mycobacterium tuberculosis-induced cell death and tissue necrosis. J. Exp. Med..

[46] Amaral E.P., Foreman T.W., Namasivayam S., Hilligan K.L., Kauffman K.D., Barbosa Bomfim C.C., Costa D.L., Barreto-Duarte B., Gurgel-Rocha C., Santana M.F., Cordeiro-Santos M., Du Bruyn E., Riou C., Aberman K., Wilkinson R.J., Barber D.L., Mayer-Barber K.D., Andrade B.B., Sher A. (2022). GPX4 regulates cellular necrosis and host resistance in Mycobacterium tuberculosis infection. J. Exp. Med..

[47] Casanova J.L., Abel L. (2002). Genetic dissection of immunity to mycobacteria: the human model. Annu. Rev. Immunol..

[48] Flynn J.L., Chan J., Triebold K.J., Dalton D.K., Stewart T.A., Bloom B.R. (1993). An essential role for interferon gamma in resistance to Mycobacterium tuberculosis infection. J. Exp. Med..

[49] Giresi P.G., Kim J., McDaniell R.M., Iyer V.R., Lieb J.D. (2007). FAIRE (Formaldehyde-Assisted isolation of regulatory elements) isolates active regulatory elements from human chromatin. Genome Res..

[50] Wei S.L., Yang Y., Si W.Y., Zhou Y., Li T., Du T., Zhang P., Li X.L., Duan R.N., Duan R.S., Yang C.L. (2023). Methylglyoxal suppresses microglia inflammatory response through NRF2-IkappaBzeta pathway. Redox Biol..

[51] Egawa T., Ogawa T., Yokokawa T., Kido K., Goto K., Hayashi T. (2022). Methylglyoxal reduces molecular responsiveness to 4 weeks of endurance exercise in mouse plantaris muscle. J. Appl. Physiol..

[52] Martinez N., Kornfeld H. (2014). Diabetes and immunity to tuberculosis. Eur. J. Immunol..

[53] Sugawara I., Mizuno S. (2008). Higher susceptibility of type 1 diabetic rats to Mycobacterium tuberculosis infection. Tohoku J. Exp. Med..

[54] Restrepo B.I., Khan A., Singh V.K., Erica d.-L., Aguillon-Duran G.P., Ledezma-Campos E., Canaday D.H., Jagannath C. (2021). Human monocyte-derived macrophage responses to M. tuberculosis differ by the host's tuberculosis, diabetes or obesity status, and are enhanced by rapamycin. Tuberculosis.

[55] Gomez D.I., Twahirwa M., Schlesinger L.S., Restrepo B.I. (2013). Reduced Mycobacterium tuberculosis association with monocytes from diabetes patients that have poor glucose control. Tuberculosis.

[56] Panda S., Seelan D.M., Faisal S., Arora A., Luthra K., Palanichamy J.K., Mohan A., Vikram N.K., Gupta N.K., Ramakrishnan L., Singh A. (2022). Chronic hyperglycemia drives alterations in macrophage effector function in pulmonary tuberculosis. Eur. J. Immunol..

[57] Brownlee M. (2005). The pathobiology of diabetic complications: a unifying mechanism. Diabetes.

[58] Sun Q., Shen X., Ma J., Lou H., Zhang Q. (2020). Activation of Nrf2 signaling by oltipraz inhibits death of human macrophages with Mycobacterium tuberculosis infection. Biochem. Biophys. Res. Commun..

[59] Dafre A.L., Goldberg J., Wang T., Spiegel D.A., Methylglyoxal P. Maher (2015). The foe and friend of glyoxalase and Trx/TrxR systems in HT22 nerve cells. Free Radic. Biol. Med..

[60] Venketaraman V., Dayaram Y.K., Talaue M.T., Connell N.D. (2005). Glutathione and nitrosoglutathione in macrophage defense against Mycobacterium tuberculosis. Infect. Immun..

[61] Rabbani N., Thornalley P.J. (2011). Glyoxalase in diabetes, obesity and related disorders. Semin. Cell Dev. Biol..

[62] Liang J., Zhang X.Y., Zhen Y.F., Chen C., Tan H., Hu J., Tan M.S. (2019). PGK1 depletion activates Nrf2 signaling to protect human osteoblasts from dexamethasone. Cell Death Dis..

[63] Zheng J., Zhu J.L., Zhang Y., Zhang H., Yang Y., Tang D.R., Sun J. (2020). PGK1 inhibitor CBR-470-1 protects neuronal cells from MPP+. Aging (Albany NY).

[64] Anestal K., Arner E.S. (2003). Rapid induction of cell death by selenium-compromised thioredoxin reductase 1 but not by the fully active enzyme containing selenocysteine. J. Biol. Chem..

[65] Xu J., Cheng Q., Arner E.S. (2016). Details in the catalytic mechanism of Mammalian thioredoxin reductase 1 revealed using point mutations and juglone-coupled enzyme activities. Free Radic. Biol. Med..

[66] Coukos J.S., Lee C.W., Pillai K.S., Liu K.J., Moellering R.E. (2023). Widespread, reversible cysteine modification by methylglyoxal regulates metabolic enzyme function. ACS Chem. Biol..

[67] Dunigan K., Li Q., Li R., Locy M.L., Wall S., Tipple T.E. (2018). The thioredoxin reductase inhibitor auranofin induces heme oxygenase-1 in lung epithelial cells via Nrf2-dependent mechanisms. Am. J. Physiol. Lung Cell. Mol. Physiol..

[68] Locy M.L., Rogers L.K., Prigge J.R., Schmidt E.E., Arner E.S., Tipple T.E. (2012). Thioredoxin reductase inhibition elicits Nrf2-mediated responses in clara cells: implications for oxidant-induced lung injury. Antioxidants Redox Signal..

[69] Berends E., van Oostenbrugge R.J., Foulquier S., Schalkwijk C.G. (2023). Methylglyoxal, a highly reactive dicarbonyl compound, as a threat for blood brain barrier integrity. Fluids Barriers CNS.

[70] Bondeson J., Sundler R. (1995). Auranofin inhibits the induction of interleukin 1 beta and tumor necrosis factor alpha mRNA in macrophages. Biochem. Pharmacol..

[71] Wall S.B., Li R., Butler B., Burg A.R., Tse H.M., Larson-Casey J.L., Carter A.B., Wright C.J., Rogers L.K., Tipple T.E. (2021). Auranofin-mediated NRF2 induction attenuates interleukin 1 beta expression in alveolar macrophages. Antioxidants.

[72] Murray H.W., Spitalny G.L., Nathan C.F. (1985). Activation of mouse peritoneal macrophages in vitro and in vivo by interferon-gamma. J. Immunol..

[73] Gao Y., Basile J.I., Classon C., Gavier-Widen D., Yoshimura A., Carow B., Rottenberg M.E. (2018). STAT3 expression by myeloid cells is detrimental for the T- cell-mediated control of infection with Mycobacterium tuberculosis. PLoS Pathog..

[74] Matic M., Simon S.R. (1992). Effects of gamma interferon on release of tumor necrosis factor alpha from lipopolysaccharide-tolerant human monocyte-derived macrophages. Infect. Immun..

[75] Chen J., Ivashkiv L.B. (2010). IFN-gamma abrogates endotoxin tolerance by facilitating toll-like receptor-induced chromatin remodeling. Proc. Natl. Acad. Sci. U. S. A..

[76] Docke W.D., Randow F., Syrbe U., Krausch D., Asadullah K., Reinke P., Volk H.D., Kox W. (1997). Monocyte deactivation in septic patients: restoration by IFN-gamma treatment. Nat. Med..

[77] Hotchkiss R.S., Monneret G., Payen D. (2013). Sepsis-induced immunosuppression: from cellular dysfunctions to immunotherapy. Nat. Rev. Immunol..

[78] Su X., Yu Y., Zhong Y., Giannopoulou E.G., Hu X., Liu H., Cross J.R., Ratsch G., Rice C.M., Ivashkiv L.B. (2015). Interferon-gamma regulates cellular metabolism and mRNA translation to potentiate macrophage activation. Nat. Immunol..

