# Supplementary material for: The inhibition of TXNRD1 by methylglyoxal impairs the intracellular control of Mycobacterium tuberculosis
Source: Redox Biol. 2025 Jun 25;85:103741. doi: 10.1016/j.redox.2025.103741 (PMC12271799; doi:10.1016/j.redox.2025.103741)
Supplement: Multimedia component 1 [file mmc1.docx]

### Supplementary figure 1**
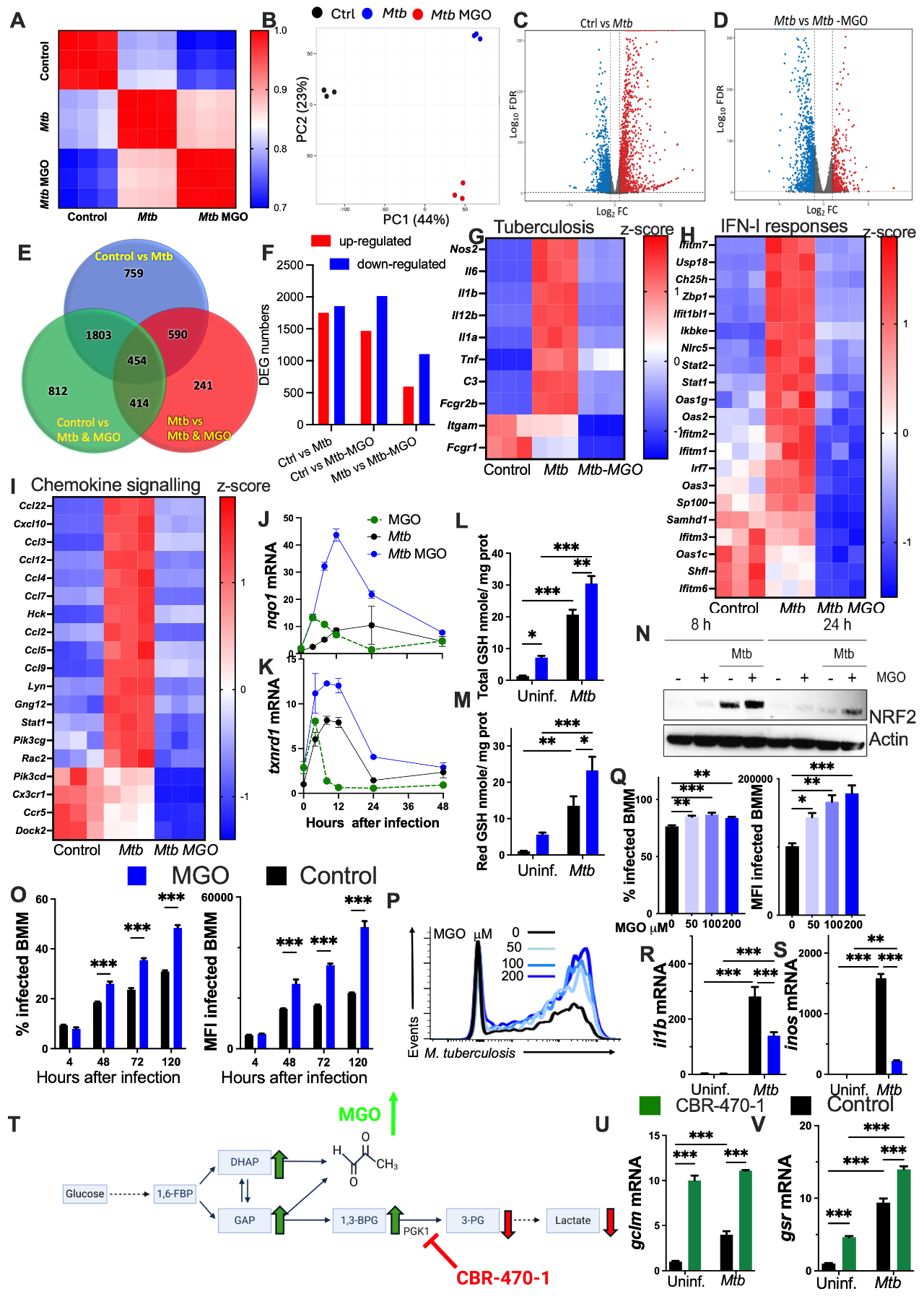
**

### MGO impairs the expression of inflammatory genes and stimulates the expression of antioxidant genes in M. tuberculosis infected BMM

**(A)** RNA seq was performed in triplicate independent cultures of BMM treated or not with 200 μM MGO and infected 4 h after with *Mtb*. RNA was extracted 4 h after infection and RNA seq performed as indicated in Methods section. The Pearson R^2^ correlation coefficient matrix of the expression level of all genes in each sample is shown as a heat map.

**(B)** PCA plots from non-supervised samples based on the normalized gene counts after filtering the low expressed genes are shown.

**(C and D)** Volcano plots showing the log_2_ fold change (the gene expression difference between both samples) in the x-axis and the statistical significance in the y-axis, representing the overall distribution of differentially upregulated or downregulated genes in uninfected vs. *Mtb*-infected and *Mtb* vs *Mtb*-MGO BMM are depicted.

**(E)** The Venn diagram represents the number of genes determine by RNA seq that are common to or uniquely expressed within non infected and *Mtb*, *Mtb* -MGO, and uninfected controls.

**(F)** The number of differentially expressed genes (up or down-regulated) for each comparison combination is shown.

**(G-I)** The heat maps of RNA-seq data showing differentially expressed genes within tuberculosis (**G**), response to IFN-I (H) and chemokine signalling (**I**) KEGGs in *Mtb* infected BMM treated or not with MGO.

**(J and K)** BMM were treated or not with MGO and 4 h after infected with *Mtb*. The levels of *nqo1* and *txnrd1* mRNA in total RNA were measured by RT-PCR extracted at the indicated time points after infection.

**(L and M)** The levels of total (GSH + GSSG) and reduced (GSH) glutathione were measured in BMM cell lysates using glutathione reductase-coupled DTNB reduction assay in a system with NADPH.

**(N)** BMM lysates were analysed by Western blot before and after *Mtb infection* and/ or MGO-treatment using anti-NRF2 and anti-β-actin antibodies.

**(O)** BMM were treated or not with 200 μM MGO and infected 4 h after with *Mtb*-GFP at a MOI of 3:1. 4 h after infection BMM were washed and further incubated for the indicated times, at which the GFP-labelling in BMM was analyzed by flow cytometry. The percentage of infected BMM and the *Mtb*-GFP MFI are shown.

(**P and Q**) BMM were treated with different concentrations of MGO and infected with *Mtb*-GFP 4 h after treatment. A representative histogram, the % of infected BMM and the MFI of *Mtb*-GFP in macrophages are shown.

**(R and S)** BMM were treated with MGO, infected 4 h after with *Mtb* and the total RNA extracted 4 h after infection. The relative concentration of *il1b* and *inos* mRNA are shown.

**(T)** Scheme depicting the increased production of MGO after inhibition of PGK-1 (adapted from ^19^).

**(U and V)** BMM were treated with 5 μM CBR-470-1 for 4 h before infection with *Mtb* and total RNA was extracted 24 h after infection. The *gclm* and *gsr* mRNA levels are depicted.

**(J-L, Q-S, U,V)** Data are mean ± SEM from n = 3 independent samples per group and time point. Differences are significant at *p≤0.05, **p≤0.01 and ***p≤0.001 two-way ANOVA **(L, M, O, R, S, U, V)** and one way ANOVA **(Q)**.

Supplementary figure 2

**
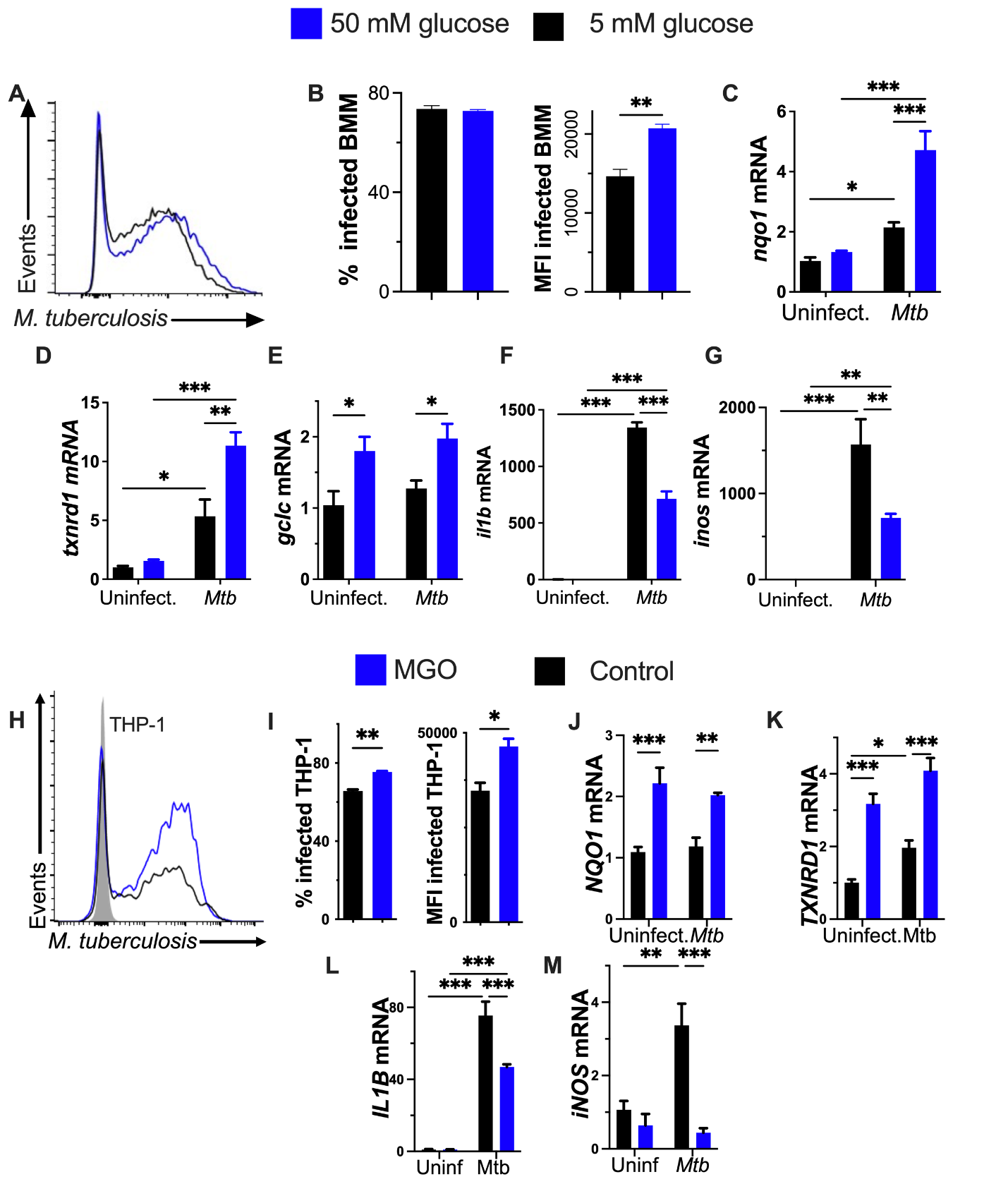
**

### High glucose levels impair the expression of inflammatory genes and stimulates the expression of antioxidant genes in M. tuberculosis infected BMM

(**A and B**). BMM were incubated with either 5 mM (low glucose/ control) or 50 mM (high glucose) starting from day 4 of differentiation and throughout the infection. A representative histogram, the percentage of infected BMM and the *Mtb-*GFP MFI in infected cells 5 days after infection are shown.

**(C-G)** BMM were treated with 50 or 5 mM glucose as described above and infected with *Mtb*. The relative levels of *nqo1, txnrd1, gclc, il1b* and *inos* mRNA were determined 24 h after infection by RT-PCR.

**(H and I)** Human THP-1 macrophages were incubated with MGO and infected 4 h after with Mtb-GFP at a MOI of 3:1. MGO was replenished 4 h after infection. Representative histograms of *Mtb*-GFP content, the percentage of infected BMM and the *Mtb-*GFP MFI in infected THP-1 at 5 days after infection are shown.

**(J-M)** THP-1 cells were treated with 200 μM MGO and infected 4 h after with *Mtb* at a MOI of 5:1. Total RNA was extracted 4 h after infection. The *NQO1, TXNRD1, IL1B* and *iNOS* mRNA were determined by RT-PCR. The fold increase of transcript levels normalized to the *HPRT* mRNA levels in the same sample and to untreated controls were determined.

(**B-G and I-M**) The mean ± SEM of independent samples (n=3 per group) are shown. Differences are significant at *p≤0.05, **p≤0.01 and ***p≤0.001, unpaired Student’s ı test with Welch correction (**B and I**) and 2-way ANOVA test (C-G). Only differences between infected groups are shown (**C-G and J-M**).

Supplementary figure 3


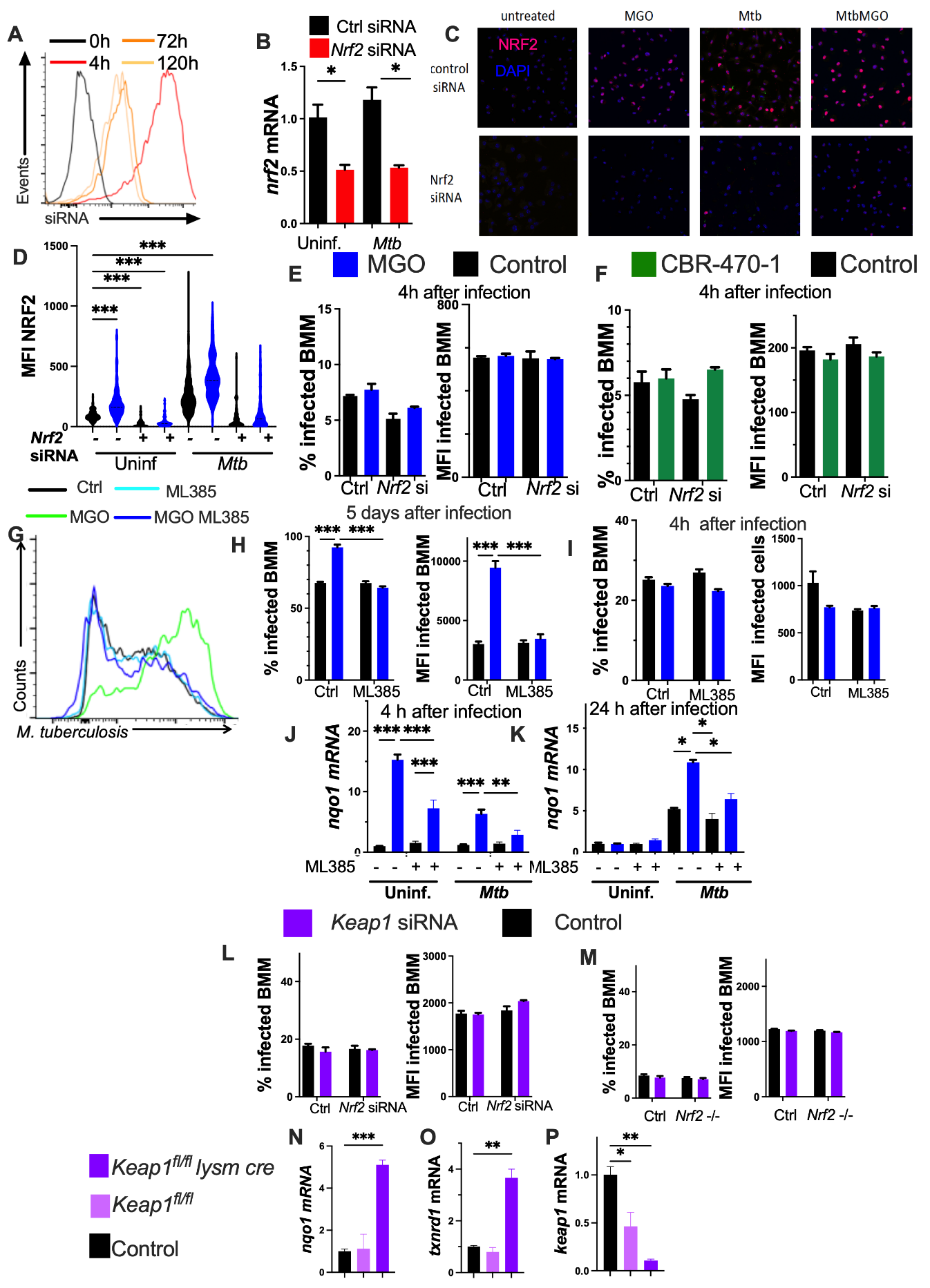


### NRF2 signalling hampers the control of intracellular M. tuberculosis in MGO-treated BMM

**(A)** BMM were labelled with 5 nM Cy5-labelled control siRNA and the transfection media replaced 4 h after labelling. The histogram depicts the levels of siRNA determined at different times after transfection of BMM.

**(B)** The mean relative levels of *nrf2* mRNA ± SEM were determined in BMM transfected with *Nrf2* or control siRNA, and infected with *Mtb* 24 h later. RNA was extracted 24 h after infection.

**(C and D)** BMM were transfected with *Nrf2* or control siRNA, treated with MGO 24 h and infected with *Mtb* (MOI 3:1) 4 h after treatment, and fixed 4 h after infection. DAPI was used for nuclear staining. Micrographs showing NRF2 immunostaining in BMM transfected with *Nrf2* or control siRNA are shown. The NRF2 label intensity in BMM was quantified using the Cell Profiler software. The fluorescence intensity of NRF2 was determined in triplicate independent samples (4 optical fields per slide). The mean NRF2 intensity per cell ± SEM in a representative sample is shown.

**(E and F)** BMM were transfected with either *Nrf2* or control siRNAs 24 h before treatment with MGO or CBR-470. 4 h after MGO treatment BMM were infected with *Mtb-*GFP. The percentage of infected BMM and the *Mtb*-GFP MFI gated on infected cells, determined 4 h after infection are shown.

**(G-I)** BMM were incubated with 200 μM MGO and/ or 5μM ML385 4 h and 1 h before infection with *Mtb*-GFP respectively. Representative histograms of GFP content 5 days after infection, the percentage of infected BMM and the *Mtb*-GFP MFI of infected BMM at 4 h and 5 days after infection are shown.

**(J and K)** BMM were treated with MGO, 3 h later with ML385 and infected with *Mtb* 1 h after. The levels of *nqo1* were determined by real time RT-PCR at 4 h and 24 h after infection.

**(L and M)** BMM were transfected with *Nrf2*, *Keap1* and/ or control siRNAs **(L)**. In other set of experiments, wild type (WT) or *Nrf2^-/-^* BMM were transfected with *keap1* or control siRNAs **(M)**. BMM were infected *Mtb-*GFP 24 h after transfection with the siRNAs. The percentage of infected BMM and the *Mtb*-GFP MFI on infected BMM 4 h after infection are shown.

**(N-P)** The fold increase of *nqo1, txnrd1, and keap1* mRNA in total RNA from WT*, Keap1^fl/fl^* and *Keap1^fl/fl^ lysm cre* BMM was determined after RT PCR 24 h after BMM differentiation.

**(B and E-P)** The mean ± SEM (n=3 independent cultures per group) are depicted. Differences determined by two-way ANOVA **(B, E, F and H-M)** and one way ANOVA **(D, N-P)** are significant at *p≤0.05, **p≤0.01 and ***p≤0.001.

## Supplementary figure 4


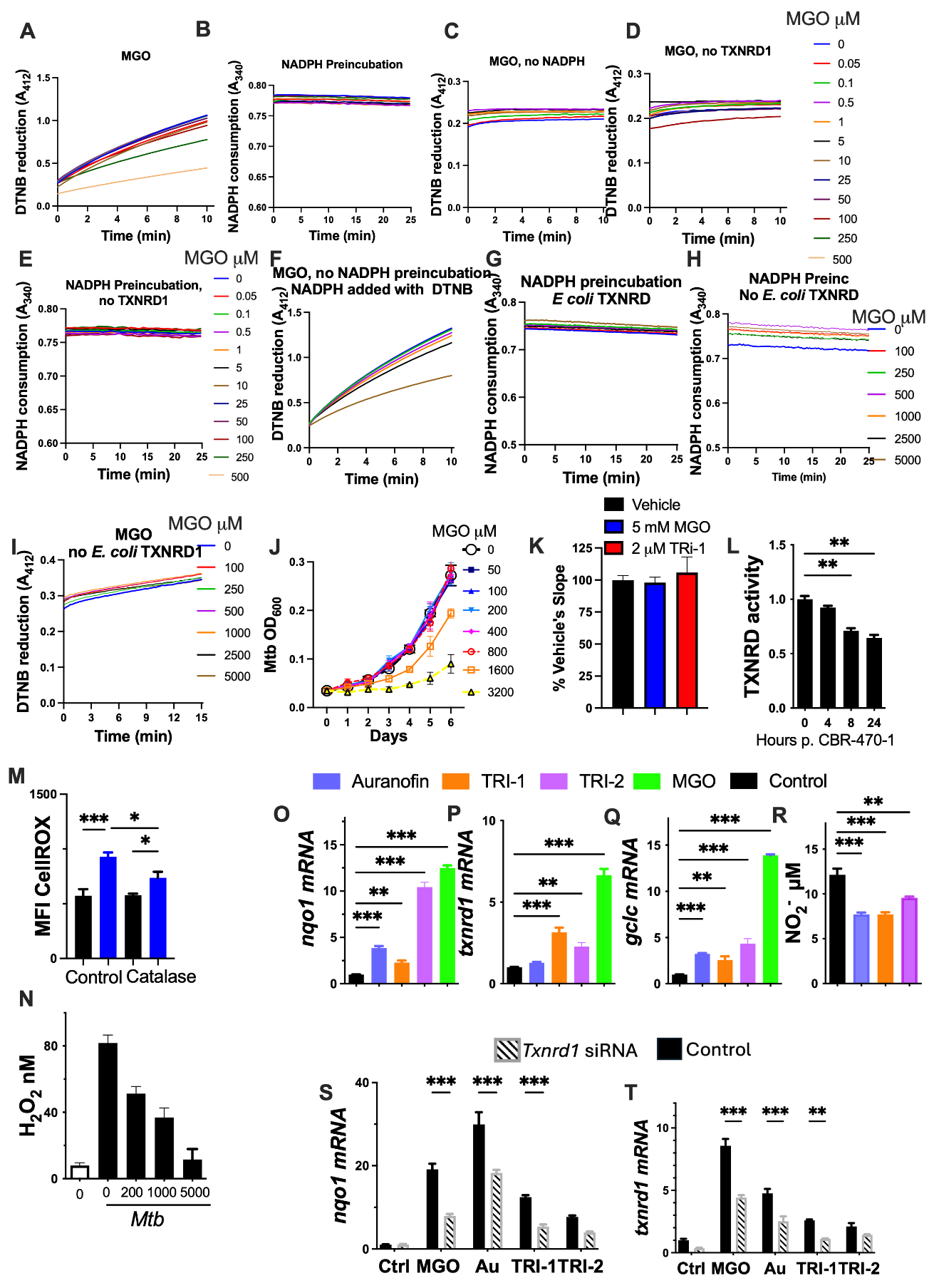


### MGO inhibits the reductase activity of TXNRD1 and converts the enzyme into a NADPH oxidase

**(A and B)** MGO was spotted at 0-500 μM and a mix of 15 nM human recombinant TXNRD1, 0.25 mM NADPH and 0.1 mg/ml BSA was added on top. The consumption of NADPH was followed for 30 min by reading at A_340_ **(B)**. Then 2.5 mM DTNB were added and the accumulation of TNB was followed at A_412_ for 10-15 min **(A)**.

**(C, D and F)** The accumulation of TNB was followed when NADPH was omitted from the reaction, when TXNRD1 was omitted from the reaction, or when NADPH was omitted during preincubation with TXNRD1 and was only added when the compound mix was incubated with DTNB.

**(E, G-I)** The NADPH oxidation was followed when TXNRD1 was lacking in the compound mix in presence of DTNB **(E)**. **(G-I)** The consumption of NADPH was also followed in the reaction between *E coli* TXNRD with MGO prior **(G)**, or after DTNB addition but in absence of *E coli* TXNRD **(H)**. In the latter case DTNB reduction was also measured at A_412_ **(I)**.

**(J)** Growth of *Mtb* cultured at 37^o^C in axenic medium measured by densitometry.

**(K)** The reaction slope of the NADPH oxidation by TXNRD1 in presence of MGO or TRI-1 when juglone was added to the reaction was compared.

**(L)** The TXNRD activity in lysates of BMM was measured by DTNB reduction at different time points after incubation with CBR-470-1. The TXNRD activity was normalized to the protein concentration of the lysates and the relative activity of each sample as compared to that of untreated/ non-infected controls.

**(M)** BMM were treated with MGO and/ or with 10^3^ U/ ml catalase. The MFI of Cell ROX evaluated 24 h after the treatment with catalase is shown.

**(N)** The BMM culture medium was replaced by PBS 48h after *Mtb* infection when the indicated catalase concentrations were added. Cultures were further incubated for 90 min when the H_2_O_2_ level in supernatants was determined by the Amplex Red assay.

**(O-Q)** BMM were treated with either 0.3 μM auranofin, 2 μM TRI-1, 3 μM TRI-2, or 200 μM MGO, and the total RNA was extracted at 8 h after treatment. The relative *nqo1*, *txnrd1* and *gclc* mRNA levels are depicted.

**(R)** BMM were treated with the TXNRD1 inhibitors and infected with *Mtb* 4 h after treatment. The nitrite levels in culture supernatants 5 days after infection.

**(S and T)** BMM were transfected with *Txnrd1* or control siRNA 24 h before incubation with either MGO, auranofin, TRI-1, or TRI-2. The relative levels of *nqo1* and *txnrd1* mRNA was determined by real time PCR.

**(L-T)** The mean ± SEM of 3 independent samples per group are shown. Differences with untreated controls are significant by one way ANOVA **(L, N-R)**, or two way ANOVA tests **(M, S and T)** at *p≤0.05, **p≤0.01 and ***p≤0.001.

Supplementary figure 5

**
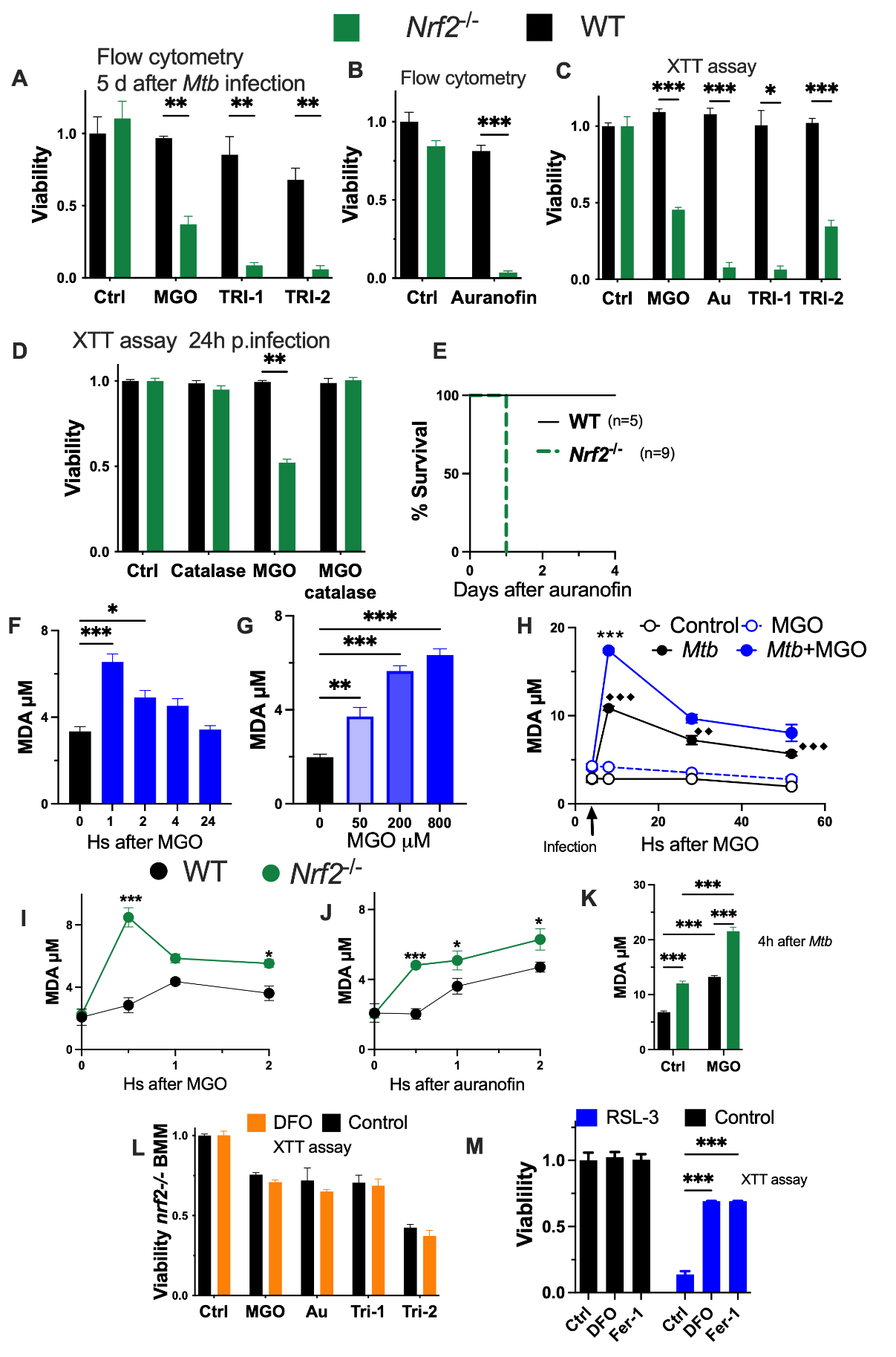
**

NRF2 activation protects BMM against MGO-generated oxidative stress

**(A and B)** WT or *Nrf2^-/-^* BMM were treated with MGO, TRI-1, TRI-2 **(A)** or auranofin **(B)** and infected with *Mtb* 4 h after treatment. The viability of BMM in cultures was evaluated by flow cytometry after 5 days of infection.

**(C)** The viability of WT or *Nrf2^-/-^* BMM was measured 24 h after treatment with MGO, TRI-1, TRI-2 or auranofin using the XTT assay, measuring XTT conversion to formazan and normalizing to respective controls.

**(D)** WT or *Nrf2^-^*^/-^ BMM were treated with MGO and / or catalase and infected 4 h after treatment. The viability of BMM was evaluated by the XTT assay 24 h after *Mtb* infection.

**(E)** The % surviving WT (n=5) and *Nrf2^-/-^*  (n=9) mice after daily i.p. inoculation with 10 mg/ kg auranofin. Differences in the survival curve are significant at 0.001, log-rank (Mantel-Cox) test.

**(F-H)** Lipid peroxidation was measured by the MDA assay in lysates from BMM treated either with different concentrations of MGO during 1 h **(F)**, at different time points after treatment with 200 μM MGO **(G)** or at different time points after *Mtb*-infected in MGO-treated cells (**H)**.

**(I-K)** MDA levels were measured in WT and *Nrf2^-/-^* BMM at the indicated times after incubation with either MGO **(I)** or auranofin **(J)**. WT and *Nrf2^-/-^* BMM were treated with MGO and infected with *Mtb* 4 h after treatment. MDA was determined 4 h after infection **(K)**.

**(L)** *Nrf2^-/-^* BMM were treated with 100 μM deferoxamine (DFO) and 1 h later incubated with either MGO, auranofin, MGO, TRI-1 or TRI-2. Viable cells were determined 24 h after treatment by XTT.

**(M)** BMM were incubated with 6 μM RSL3 and 1 h later cells were treated either with 100 μM DFO or 10 μM ferrostatin-1. The viability of RSL-3 treated or control BMM were determined by XTT 24 h after incubation with the compounds.

**(A-D, F-M)** The mean ± SEM of triplicate independent cultures are shown. Differences with MGO-treated and untreated cultures are significant at *p≤0.05, **p≤0.01 and ***p≤0.001, one way ANOVA, two-way ANOVA or multiple unpaired *t* tests. Differences with similarly treated WT controls are shown (**A-D, I and J**). Differences with *Mtb**or with control ◆ groups are significant **(H)**.

Supplementary figure 6


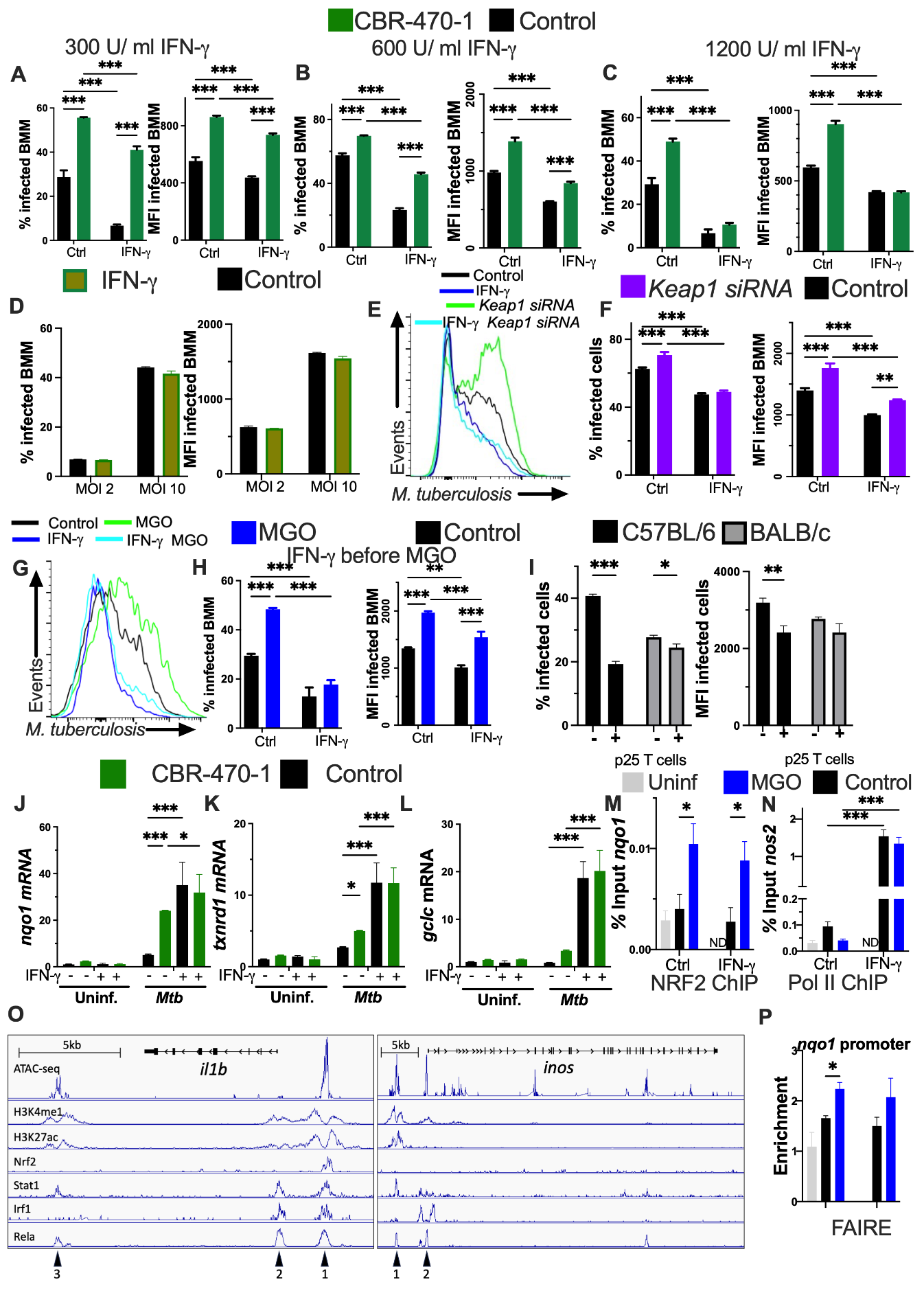


### Interferon-γ abrogates the MGO-mediated suppression of protective macrophage anti M. tuberculosis responses.

**(A-C)** BMM were incubated with the indicated concentrations of recombinant IFN-γ, treated with CBR-470-1 48 h later and infected with *Mtb*-GFP 4 h after the treatment. IFN-γ was replenished 4 h after infection. The percentage of infected BMM and the *Mtb*-GFP MFI gated on infected BMM 5 days after infection are shown.

**(D)** BMM were incubated with 1200 U/ ml IFN-γ and infected with different MOI of *Mtb* 48 h later. The fraction of infected BMM and the *Mtb*-GFP MFI in infected cells were determined 4 h after infection.

**(E and F)** BMM were incubated with IFN-γ, transfected with *Keap1* siRNA or control siRNA 4 h after and infected with *Mtb* 48 h after transfection. IFN-γ was replenished 4 h after the infection. A representative histogram, the percentage of infected BMM and the *Mtb*-GFP MFI gated on infected cells were determined 5 days after infection.

**(G and H)** BMM were incubated with 1200 U/ml IFN-γ, treated with MGO 48 h later and infected with *Mtb*-GFP 4 h after the treatment. IFN-γ was not replenished after infection. A representative histogram, the percentage of infected BMM and the *Mtb*-GFP MFI gated on infected cells were determined 5 days after infection.

**(I)** C57BL/6 (H2^b^) or BALB/c (H2^d^) BMM were infected with *M. tuberculosis*. 4 h after the infection, BMM were co-cultured with p25 transgenic (H2^b^) CD4 T cells. The percentage of infected BMM and the *Mtb*-GFP MFI in infected cells determined 5 days after infection is depicted.

**(J-L)** BMM were incubated with IFN-γ, treated with CBR-470-1 48 h later and infected with *Mtb* 4 h after treatment. Total mRNA was isolated from BMM 24 h after infection. The relative levels of *nqo1*, *txnrd1* and *gclc* mRNA were determined by RT-PCR.

(**M-N)** BMM were stimulated with IFN-γ and 24 h after treated with MGO. 4 h after treatment, BMM were infected with *M. bovis* BCG. The binding of NRF2 to *nqo1* (at different site than those in Fig. 5N) and of RNA polymerase II to *inos* genes were measured in BMM lysates by ChIP-qPCR. The % input is depicted.

**(O)** Genome tracks displaying normalized profiles for ATAC-seq and ChIP-seq signals at *il1b* and *inos* genes in macrophages. The treatments, accession numbers of the repositories and the reference’s PMID are shown in Supplementary table 5.

**(P)** BMM were treated for 4 h with MGO before infection with *M. bovis* BCG. 4 h after the quantification of open chromatin at a region including the promoter of *nqo1* gene was evaluated using the FAIRE assay.

**(A-N and P)** The mean ± SEM of 3 independent samples per group is shown.

Differences are significant at *p≤0.05, **p≤0.01 and ***p≤0.001 2-way ANOVA test.

Supplementary figure 7


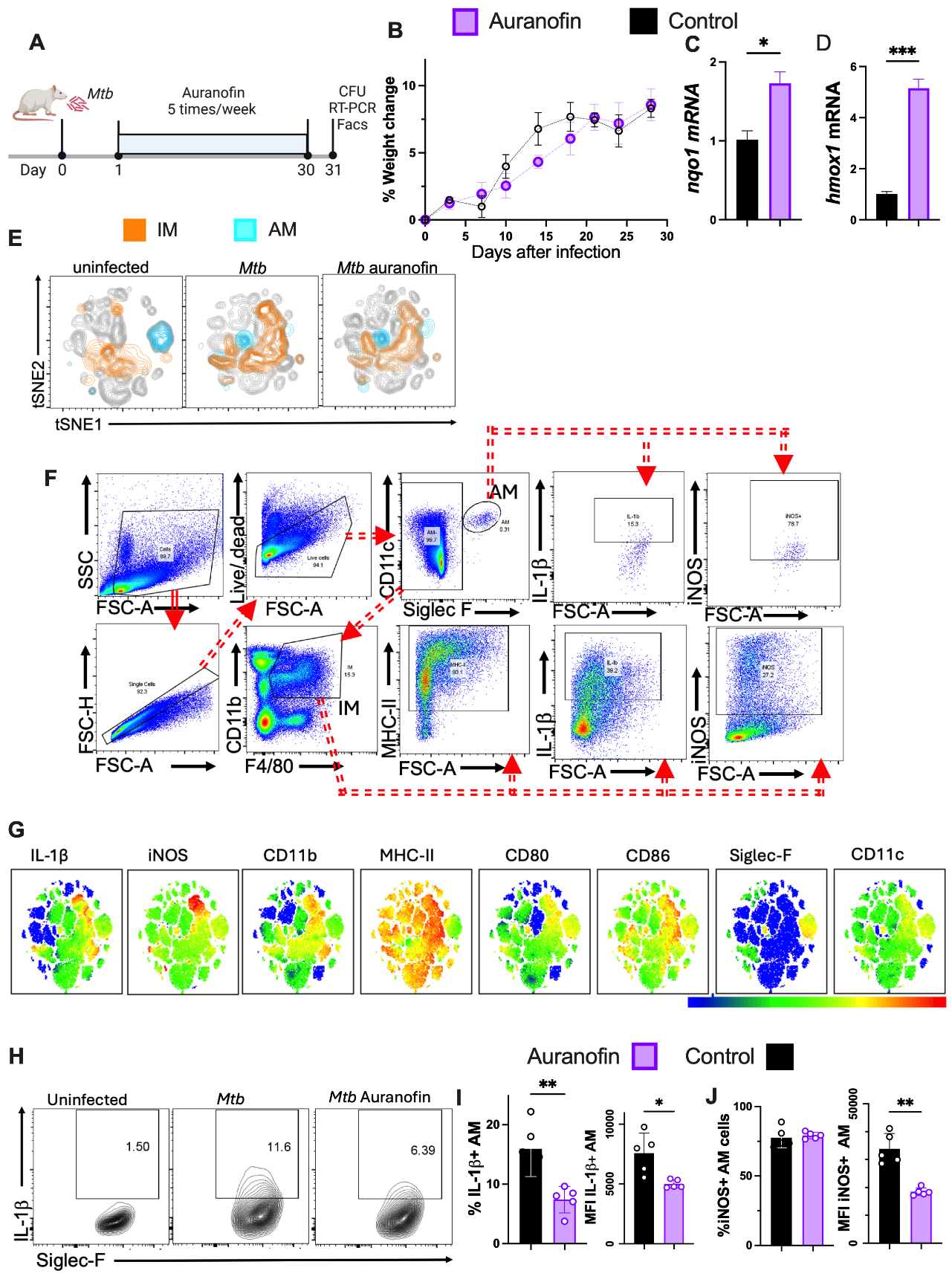


### Administration of auranofin increased NRF2 downstream responses and impaired IL-1β and iNOS production in lung macrophages from M. tuberculosis-infected mice

**(A)** Mice were administered i.p. with 10 mg/ kg auranofin daily (five days per week) during 4 weeks, starting the day after infection with *Mtb*. The animals were sacrificed 24 h after the last dose of auranofin.

**(B)** The % weight change of each animal at different time points after *Mtb* in relation to its weight before infection was determined. The mean % weigh change (n=8 per group) ± SEM in auranofin treated and control mice are depicted.

**(C and D)** Mice were treated once daily for 2 days with auranofin 10 mg/ kg. The accumulation of *nqo1* and *hmox1* mRNA in lungs was evaluated by RT-PCR 24 h after the last auranofin dose. Differences are significant at *p≤0.05 and ***p≤0.001 unpaired *t* test with Welch’s correction.

**(E)** Events in the F4/80 gate from lung cell suspensions from infected/ treated and control animals were concatenated and down-sampled to 0.15 million cells. The tSNE representation of concatenated F4/80+ lung cells from uninfected, *Mtb* and *Mtb*-auranofin treated groups, in which alveolar (AM) and inflammatory macrophages (IM) defined in a conventional manual were projected in the tSNE space and assigned different colours are shown.

**(F)** The conventional FACS gating strategy for analysing lung alveolar and inflammatory macrophages and the expression of iNOS and IL-1β is shown using a *Mtb* infected sample as example.

**(G)** The expression of different markers were then integrated into a tSNE analysis. tSNE representation of concatenated F4/80+ lung cells from uninfected, *Mtb* and *Mtb*-auranofin treated groups (as described in pannel A), in which alveolar (AM) and inflammatory macrophages (IM) defined in a conventional manual analysis were projected in the tSNE space and assigned different colours are shown.

**(H-J)** Contour plots for IL-1β from concatenated samples **(H)**, the mean frequencies and the MFI ± SEM of IL-1β **(I)** and iNOS **(J)** in alveolar macrophages (AM). AM were gated as F4/80+SiglecF+CD11c+ cells in the lungs of mice (n=5 per group) treated or not with auranofin one month after infection with *Mtb*. Differences are significant at *p≤0.05, **p≤0.01 and ***p≤0.001 unpaired t test with Welch’s correction.

## Supplementary Table 1

### List of RT-PCR primers used for mouse BMM

| **Targets** | **Forward** | **Reverse** | |
| --- | --- | --- | --- |
| *hprt* | CCCAGCGTCGTGATTAGC | GGAATAAACACTTTTTCCAAATCC |  |
| *il1β* | TGGTGTGTGACGTTCCCATT | CAGCACGAGGCTTTTTTGTTG |  |
| *inos* | CAGCTGGGCTGTACAAACCTT | CATTGGAAGTGAAGCGTTTCG |  |
| *nqo1* | TTCCCATTGCAGTGGTTTGG | CTGCTACGAGCACTCTCTCA |  |
| *gclc* | CCTGGAGCCTCTGAAGAACA | GCCAGAAGAT GATCGATGCC |  |
| *gclm* | TGAGGTACTCGGTCATCGTG | GTTCCAGACAACAGCAGGTC |  |
| *gsr* | CAGATGTTGACTGCCTGCTC | GACGTCTCCCACAGCATAGA |  |
| *hmox1* | TGTCTGAGGCCTTGAAGGAG | CAGGGCCGTGTAGATATGGT |  |
| *txnrd1* | TGAGAATGCTTACGGGAGGT | AGGAACCGCTCTGCTGAATA |  |
| *nrf2* | TCCCAGCAGGACATGGATTT | GGCCTTCTCCTGTTCCTTCT |  |
| *keap1* | CAGACAGGGCATCTTGCTTC | CAGCGTCAGTTCAAGGTCTG |  |
| *glo1* | TGTGGTCTTCCCTGCTGACA | ATAGCTGTGTAGCGCTTGGC |  |

### List of RT-PCR primers used for human macrophages

| **Targets** | **Forward** | **Reverse** | |  |
| --- | --- | --- | --- | --- |
| *HPRT* | CTTCCTCCTCCTGAGCAGTC | | GCAAGACGTTCAGTCCTGTC | |
| *IL1B* | AGCTGAGGAAGATGCTGGTT | | GTGATCGTACAGGTGCATCG | |
| *INOS* | TGTGCTCTTTGCCTGTATGC | | CTGTCGCAAAGAGGATGGTG | |
| *NQO1* | CTGGCTCACTCAGAGAGGAC | | CATGGCATAGAGGTCCGACT | |
| *TXNRD1* | TGGTGAATAGAAGGCAGGCA | | GTTGTTCCATCACCGCCTAC | |

## Supplementary table 2

### List of siRNA used

## Supplementary table 3

### List of PCR primers used for ChIP-PCR

**PCR primers used for Poll ChIP**

| **Target** | | **Forward** | **Reverse** | |  |
| --- | --- | --- | --- | --- | --- |
| *Il1b* | CTCTAGTTCAGGGCAGGCAT | | | AAGGGCCACTTGACTCCAAA | |
| *Inos* | TGTGACCTAGTGTCCTTGGC | | | CTCAAGTCCTGGCCACCTTT | |
| *Nqo1* | CTTGACCAAGGCACACACAT | | | CATCACTTGGGTGCCAGAAG | |

**PCR primers used for NRF2 ChIP**

| **Target** | **Forward** | | **Reverse** | |  |
| --- | --- | --- | --- | --- | --- |
| *Il1b* | | TGATGATGTTGGCAAAGGAA | | AAAAGCTAGAGTGCCCGTCA | |
| *Nqo1-pos 1* | | AGAGACTTGTCCTCGTGTGA | | GTGTGTATACCCAGGGAGCA | |
| *Nqo1-pos 2* | | TTCAGGCTTCAGGTCCTCTG | | TGTGCTGTAGTCACGGCTAT | |

**PCR primers used for FAIRE assay**

| **Target** | **Forward** | | **Reverse** | |  |
| --- | --- | --- | --- | --- | --- |
| *Il1b-pos1* | | AAAGAGGAAGTCGGCAAAGC | | GTGCTGAGTGAGTCAAGAGC | |
| *Il1b-pos3* | | ATTGCTTGGCTTGCTTCCTC | | ACGGTTGTGTAATGCAGCTT | |
| *Inos-pos1* | | TTCAGGCTTCAGGTCCTCTG | | TGTGCTGTAGTCACGGCTAT | |
| *Inos-pos2* | | GCCAGGGTATGTGGTTTAGC | | TAGGCAGCACGTAGTCACTT | |
| *Nqo1* | | AGAGACTTGTCCTCGTGTGA | | GTGTGTATACCCAGGGAGCA | |

## Supplementary table 4

### List of antibodies used

| **Primary antibodies**  **for FACS** | | **Fluorochrome** | | **Clone** | | **Source** | |
| --- | --- | --- | --- | --- | --- | --- | --- |
| CD16/CD32 |  | | 2.4G2 | | BD | |  |
| Siglec-F | APC/Cyanine7 | | S17007L | | Biolegend | |  |
| F4/80 | Super Bright 780 | | BM8 | | eBioscience™ | |  |
| CD11c | Brilliant Violet 711 | | N418 | | Biolegend | |  |
| CD11b | Alexa Fluor 700 | | M1/70 | | eBioscience™ | |  |
| CD80 | PE/Cyanine7 | | 16-10A1 | | Biolegend | |  |
| CD86 | PE/Cyanine5 | | GL-1 | | Biolegend | |  |
| MHC II | BV421 | | M5/114.15.2 | | BD | |  |
| iNOS | PE | | CXNFT | | eBioscience™ | |  |
| IL-1β | APC | | NJTEN3 | | eBioscience™ | |  |
| CD45.1 | Brilliant Violet 711 | | 104 | | Biolegend | |  |
| CD45.2 | V450 | | A20 | | BD | |  |
| CD4 | Brilliant Violet 786 | | GK1.5 | | BD | |  |
| CD3ε | eFlour450 | | 17A2 | | eBioscience™ | |  |
| CD8a | Alexa Fluor 700 | | 3B5 | | eBioscience™ | |  |
|  |  | |  | |  | |  |
| **Primary antibodies for WB** |  | |  | |  | |  |
| NRF2 |  | | D1Z9C | | Cell Signalling | |  |
| γβ-actin |  | | A5441 | | Sigma | |  |
|  |  | |  | |  | |  |
| **Secondary conjugated antibodies** | | |  | |  | |  |
| anti-rabbit IgG | HRP-conjugated | | polyclonal | | Abcam | |  |
| anti-rabbit IgG | Rhodamine red | | polyclonal | | Jackson ImmunoResearch | |  |

## Supplementary table 5

### Atac-seq and Chip-seq data sets

| **Sequencing Dataset** | **Treatment** | **Accession number** | **Reference**  **PMID** |
| --- | --- | --- | --- |
| ATAC-seq | LPS + IFN-γ | GSM4792023 | 32703960 |
| H3K4me1 | no treatment | GSM2060962 | [27001747](https://pubmed.ncbi.nlm.nih.gov/27001747/) |
| H3K27ac | no treatment | GSM940902 | 23332752 |
| Nrf2 | diethylmaleate | GSM1944620 | [26677805](https://www.ncbi.nlm.nih.gov/pubmed/26677805) |
| Stat1 | IFN-γ | GSM3565020 | [32265223](https://www.ncbi.nlm.nih.gov/pubmed/32265223) |
| Irf1 | LPS | GSM1563687 | 25637355 |
| Rela | Kdo2 LIPID A | GSM2095114 | 28041958 |
